# Identifying structural and dynamic changes during the Biliverdin Reductase B catalytic cycle

**DOI:** 10.3389/fmolb.2023.1244587

**Published:** 2023-08-14

**Authors:** Eunjeong Lee, Matthew J. McLeod, Jasmina S. Redzic, Barbara Marcolin, Robert E. Thorne, Pratul Agarwal, Elan Zohar Eisenmesser

**Affiliations:** ^1^ Department of Biochemistry and Molecular Genetics, School of Medicine, University of Colorado Denver, Aurora, CO, United States; ^2^ Laboratory of Atomic and Solid-State Physics, Cornell University, Ithaca, NY, United States; ^3^ Department of Physiological Sciences and High Performance Computing Center, Oklahoma State University, Stillwater, OK, United States

**Keywords:** NMR, allostery, BLVRB, reductase, dynamics

## Abstract

Biliverdin Reductase B (BLVRB) is an NADPH-dependent reductase that catalyzes the reduction of multiple substrates and is therefore considered a critical cellular redox regulator. In this study, we sought to address whether both structural and dynamics changes occur between different intermediates of the catalytic cycle and whether these were relegated to just the active site or the entirety of the enzyme. Through X-ray crystallography, we determined the apo BLVRB structure for the first time, revealing subtle global changes compared to the holo structure and identifying the loss of a critical hydrogen bond that “clamps” the R78-loop over the coenzyme. Amide and Cα chemical shift perturbations were used to identify environmental and secondary structural changes between intermediates, with more distant global changes observed upon coenzyme binding compared to substrate interactions. NMR relaxation rate measurements provided insights into the dynamic behavior of BLVRB during the catalytic cycle. Specifically, the inherently dynamic R78-loop that becomes ordered upon coenzyme binding persists through the catalytic cycle while similar regions experience dynamic exchange. However, the dynamic exchange processes were found to differ through the catalytic cycle with several groups of residues exhibiting similar dynamic responses. Finally, both local and distal structural and dynamic changes occur within BLVRB that are dependent solely on the oxidative state of the coenzyme. Thus, through a comprehensive analysis here, this study revealed structural and dynamic alterations in BLVRB through its catalytic cycle that are not simply relegated to the active site, but instead, are allosterically coupled throughout the enzyme.

## Introduction

The development of solution strategies that probe macromolecular motions has revealed intimate relationships between structure and dynamics that are particularly important for enzymes, as they often rely on conformational changes for catalytic function. Some of the most important findings from nuclear magnetic resonance (NMR) relaxation experiments within the last 20 years has been that active sites of enzymes have evolved to undergo motions on timescales similar to their catalytic rates and these motions are allosterically coupled to distal sites (Lisi and Loria, 2017). Such inherent plasticity includes examples from nearly every enzyme type, such as kinases (Iverson et al., 2020), phosphatases (Whittier et al., 2013; Cui et al., 2019), proteases (Eisenmesser et al., 2015), nucleases (Narayanan et al., 2017; Narayanan et al., 2018), and oxidoreductases that are the focus of this study (Boehr et al., 2006b). Oxidoreductases represent the largest of the six major enzyme families, which in turn comprise one of the largest classes of enzyme folds referred to as short-chain dehydrogenases/reductases (SDRs) (Kavanagh et al., 2008). While such enzymes are widely reliant on their coenzymes for the functional transfer of a hydride (NADPH/NADP^+^ and NADH/NAD^+^), even the oxidation state of the coenzyme may dictate global folding and dynamics. For example, largescale spectral changes induced by a coenzyme’s oxidative state have been identified for *Rhodospirillum rubrum* transhydrogenase (Quirk et al., 1999) and the oxidative state of the coenzyme dictates its stereochemistry bound to ferredoxin (Kean et al., 2017). Identifying the changes between catalytic intermediates is often difficult, as similar X-ray crystal structures can be observed despite largescale spectral changes observed via NMR that do support global changes in solution (Quirk et al., 1999; Sundaresan et al., 2003). One explanation could be that coenzymes or substrates are often soaked into preformed crystals, yet another explanation is that the ground state observed in some crystals may not reflect changes to dynamically sampled conformations in solution. Consistent with dynamically imparted differences, the anisotropic B-factors are different between crystallized forms of NADPH and NADP^+^ bound to ferredoxin (Kean et al., 2017) and changes to conformational sampling in solution have been proposed for catalytically important active site loops within multiple oxidoreductases that include the “D loop” of *Rhodospirillum rubrum* transhydrogenase (Quirk et al., 1999) and the “M20 loop” of dihydrofolate reductases (DHFR) (Boehr et al., 2006a; Boehr et al., 2006b). In this report, we have monitored both the backbone changes and dynamic changes of a prototypical SDR family member, Biliverdin Reductase B (BLVRB), in order to determine whether there are global structural/dynamic changes through its catalytic cycle and whether its coenzyme’s hydride alone may dictate global coupling.

**FIGURE 1:**
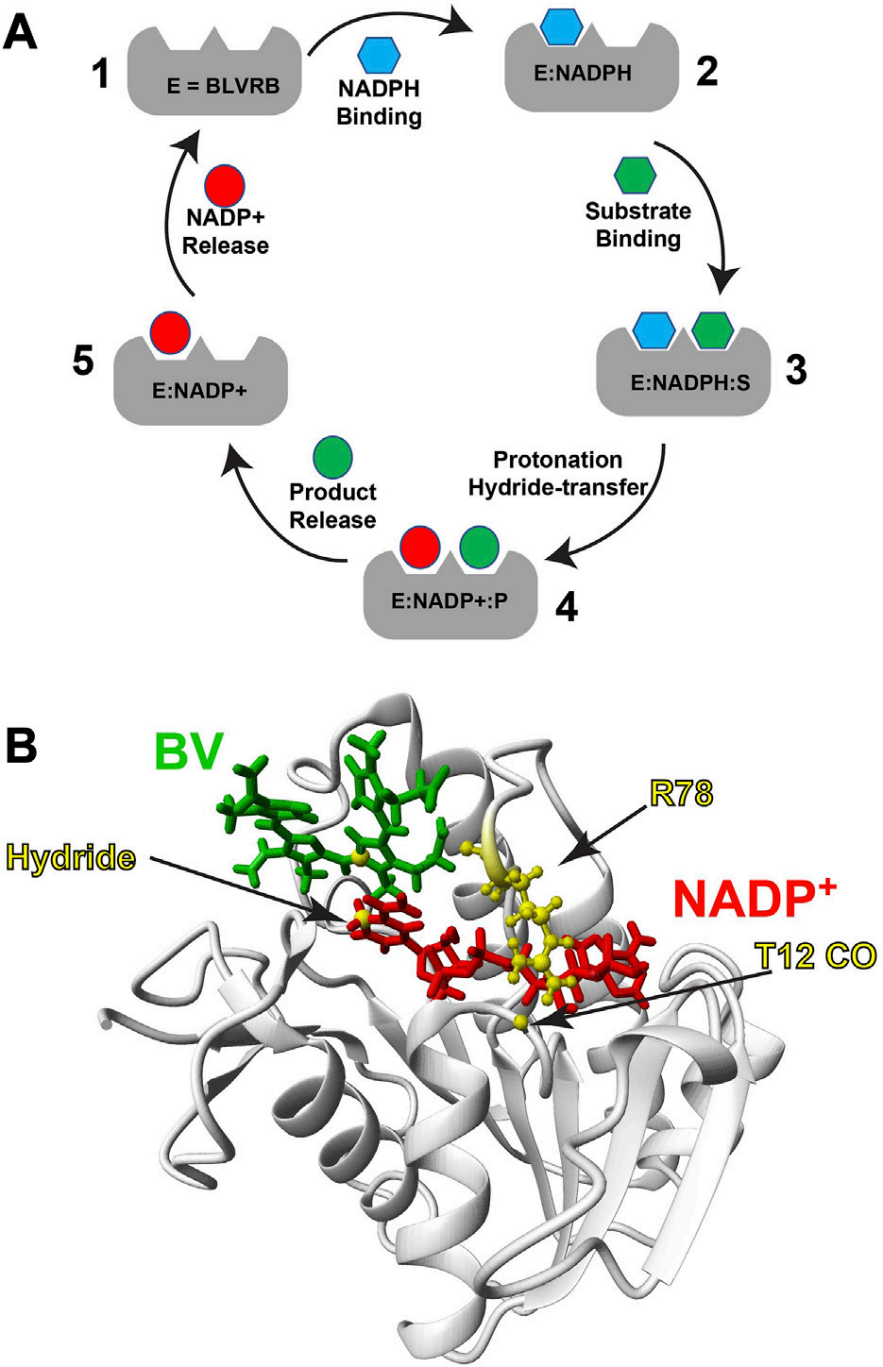
BLVRB catalytic cycle and ternary complex. **(A)** The BLVRB catalytic cycle. Apo BLVRB (1) binds NADPH (blue hexagon) to form E:NADPH (2). Substrate binding (S, green hexagon) forms the E:NADPH:S Michaelis-Menten complex (3). Reduction of the substrate results in the product (P, green circle) and oxidized NADP^+^ (red circle) to form the product complex (4). Release of the product then forms the spent coenzyme complex (5), which is released to restart the catalytic cycle. **(B)** Model of substrate bound ternary complex of BLVRB based on the X-ray crystal structure of BLVRB bound to NADP^+^ and mesobiliverdin IV (PDB accession 1HE3). Biliverdin (green), NADP^+^ (red) and R78 are highlighted.

BLVRB is an NADPH-dependent oxidoreductase that has emerged as a critical redox regulator within the last decade, as it catalyzes reduction of multiple substrates (Figure 1A). BLVRB was originally thought to reduce only the biliverdin-β isomer that is less abundant than the biliverdin-α isomer catalyzed by the BLVRA isoform (Cunningham et al., 2000; McDonagh, 2001). However, it was later discovered that BLVRB is identical to flavin reductase (FR) that is a promiscuous enzyme responsible for reduction of flavin substrates (Shalloe et al., 1996), which includes flavin adenine dinucleotide (FAD) and flavin mononucleotide (FMN). BLVRB plays such a critical role in redox regulation that this enzyme alone dictates hematopoietic cell fate (Wu et al., 2016). BLVRB-mediated redox regulation may explain why its expression is high in cancer cells. For example, BLVRB is a driver of hepatic cancer cell proliferation (Huan et al., 2016) and is a top prognostic factor for fatal prostate cancer (Ramberg et al., 2021). We have also shown that BLVRB is expressed at much higher levels in red blood cells than previously thought where it likely also plays a role in redox regulation (Paukovich et al., 2018). Biochemically, the rate-limiting step in BLVRB activity has remained unknown since its discovery, yet our biochemical and biophysical studies suggest that the slowest step identified thus far, coenzyme release, is coupled to a rate-limiting step (Duff et al., 2020; Redzic et al., 2021) (Figure 1A). Structurally, BLVRB comprises a typical SDR fold with a central β-sheet flanked α-helices on each side (Figure 1B). Like other SDR family members, the coenzyme binding site is within the conserved SDR fold itself, while substrate specificity is dictated by the variable C-terminal lobe. X-ray crystallography has revealed that BLVRB R78 forms a hydrogen bond to T12 CO that serves to straddle the coenzyme (PDB accession 1HDO), while NMR has shown that this loop, referred to as the “R78-loop”, is highly dynamic in the absence of the oxidized coenzyme (Paukovich et al., 2018). Unfortunately, it has been difficult to identify changes within BLVRB that may occur through its catalytic cycle, as only holo BLVRB has been crystalized with no apparent changes upon substrate binding (Pereira et al., 2001). Our own X-ray crystallographic studies of a human holo BLVRB mutant and even its lemur homologue that comprises 16 changes has also crystallized identically (Duff et al., 2020). Thus, here we sought to determine whether we could identify structural and dynamic changes within human BLVRB through its catalytic cycle and whether these were relegated to the active site or globally coupled sites.

## Results

### Apo BLVRB crystallizes in the same conformation as holo BLVRB

**FIGURE 2:**
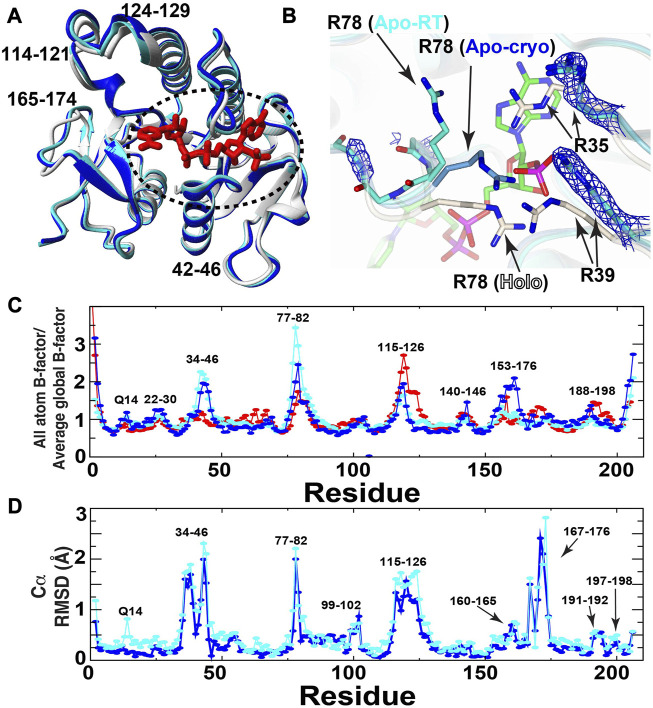
X-ray crystal structures of apo BLVRB and comparison to holo BLVRB. **(A)** Comparison of holo BLVRB bound to NADP^+^ (PDB accession 1HDO, white) to apo-cryo BLVRB (blue) and apo-RT BLVRB (cyan). The bound NADP^+^ in holo BLVRB is shown for orientation (red). Dashed circle indicates the region shown further in **(B)**. **(B)** Comparison of holo BLVRB aligned to apo-cryo BLVRB (light blue) and apo-RT BLVRB (cyan). The bound NADP^+^ in holo BLVRB is shown for orientation (green). 2F_o_-F_c_ electron density map (blue - 1.5σ) is present for indicated arginine side chain and residues 77–80 main-chain (mostly absent). While the Arg78 side chain is visible within the apo-cryo BLVRB structure at 2F_o_-F_c_ electron density of 0.9σ, this side chain position here is unobservable and deduced from the backbone. All other atoms are colored by type: oxygen—red, nitrogen—blue, phosphorous—purple. **(C)** Normalized B-factors are shown for each structure (1HDO—red). **(D)** Cα RMSDs are shown between apo BLVRB-cryo/holo BLVRB (blue) and between apo BLVRB-RT/holo BLVRB (cyan).

In an effort to identify the conformational changes within BLVRB necessary to facilitate coenzyme binding, we have solved the first X-ray crystal structures of apo human BLVRB that has eluded crystallization until now (Figure 2A). Apo BLVRB crystallized within the same space group of P2_1_2_1_2_1_ as holo BLVRB (PDB accession 1HDO) at both cryogenic (apo-cryo) and room temperature conditions (apo-RT) (Table 1). At the cofactor binding site, three arginines show clear remodeling compared to the holo state (Figure 2B). In both apo-RT and apo-cryo structures, R35 and R39 are both found in specific conformations, as indicated by clear electron density. Most surprising is that in the apo-cryo structure, the R78-loop (residues 77–80), which exhibits high R1 relaxation rates indicative of disorder in solution (Paukovich et al., 2018), is well modelled in electron density where the R78 sidechain interacts with its crystallographic mate (not shown). Although this suggests that the R78-loop is “closed” and would therefore block binding and release of the coenzyme, the R78 side chain does not form the hydrogen bond to T12 CO that forms the clamp. To determine if this defined conformation of the R78-loop is an artifact of cooling to cryogenic temperatures, the complementary room-temperature (20 °C) structure was compared. This ambient temperature structure indicates that R35 and R39 are found in the same position as the apo-cryo dataset, suggesting these conformational changes are likely important in cofactor binding. In contrast, the R78 sidechain and loop electron density is relatively absent (Figure 2B), indicating flexibility in agreement with previous NMR data (Paukovich et al., 2018) and illustrated here by B-factor comparisons (Figure 2C). Furthermore, this increased flexibility of the R78-loop would allow for coenzyme binding and release, but only as observed within the apo-RT BLVRB structure. Elsewhere, both apo structures are generally similar, but there are significant regions throughout the enzyme which differ when comparing the apo-structures and the previously determined holo-structure (0.8 Å all-atom RMSD). Specifically, loops 34–46, 115–126, and 153–176 show large displacements and conformational changes with some regions having greater than a 2 Å Cα RMSD (Figure 2D). Interestingly, these same regions are inherently dynamic on multiple timescales in solution and exhibit chemical shift perturbations (CSPs) upon coenzyme engagement (Paukovich et al., 2018), which is also further illustrated by our NMR studies below. Thus, these conformational changes observed when comparing both apo BLVRB structures to the holo-structure indicates partial remodeling of BLVRB global and active site structure upon cofactor binding, and furthermore highlight how ambient temperature experiments may better match the flexibility observed within solution NMR studies. To further identify the regions of BLVRB that change during the catalytic cycle and associated changes to motions after cofactor binding, we turned to NMR solution studies.

**TABLE 1:** X-ray data and model statistics for both Apo-RT and Apo-cryo BLVRB structures.

	Apo-RT BLVRB (293K)	Apo-cryo BLVRB (cryogenic)
Wavelength	1 Å	1 Å
Resolution range	50.5 - 2.19 (2.27 - 2.19)	52.1 - 1.52 (1.57 - 1.52)
Space group	P 21 21 21	P 21 21 21
Unit cell	42.5909 47.309 101.075 90 90 90	42.1866 45.6677 104.213 90 90 90
Total reflections	136451 (13209)	388603 (29078)
Unique reflections	10968 (1065)	31069 (2891)
Multiplicity	12.4 (12.4)	12.5 (10.1)
Completeness (%)	99.3 (97.4)	97.8 (92.5)
Mean I/sigma(I)	8.24 (1.81)	12.0 (1.40)
Wilson B-factor	33.6	14.3
R-merge	0.288 (1.296)	0.115 (1.027)
R-meas	0.301 (1.352)	0.120 (1.085)
R-pim	0.086 (0.379)	0.034 (0.338)
CC1/2	0.969 (0.972)	0.999 (0.743)
CC*	0.992 (0.993)	1 (0.923)
Reflections used in refinement	10965 (1063)	31059 (2889)
Reflections used for R-free	545 (62)	1561 (147)
R-work	0.176 (0.205)	0.159 (0.223)
R-free	0.227 (0.285)	0.187 (0.256)
Number of non-hydrogen atoms	1639	2015
macromolecules	1589	1687
ligands	1	2
solvent	48	326
Protein residues	205	205
RMS(bonds)	0.002	0.008
RMS(angles)	0.43	0.98
Ramachandran favored (%)	97.0	99.0
Ramachandran allowed (%)	2.46	0.49
Ramachandran outliers (%)	0.49	0.49
Rotamer outliers (%)	0.58	0.54
Clashscore	0.62	2.63
Average B-factor	39.7	18.7
macromolecules	39.6	16.6
ligands	33.6	16
solvent	41.4	29.8
Number of TLS groups	3	6

### Secondary structure changes between intermediates reveal global changes

In order to identify changes within BLVRB through multiple stages of its catalytic cycle, we assigned the backbone of all possible stable complexes, which included both the apo BLVRB and bound NADP^+^ complex previously published (Paukovich et al., 2018), the bound NADPH complex here, and the product ternary complex with both NADP^+^ and bilirubin. While BLVRB catalyzes reduction of multiple flavins, their affinities are in the high micromolar, and saturating conditions are not possible. Therefore, the bilirubin product was used instead, as its affinity is within the low micromolar range and would avoid contributions from exchange for relaxation experiments described below. However, commercial preparations of bilirubin used for these studies do comprise mixtures of isoforms that all bind BLVRB (Cunningham et al., 2000). We note that the Michaelis-Menten complex could not be probed here due to the obvious fact that turnover would occur within the NMR tube (with both bound NADPH and biliverdin substrate). Thus, chemical shift assignments have now been expanded to the four possible catalytic intermediates of 1, 2, 4, and 5 (see Figure 1A).

**FIGURE 3:**
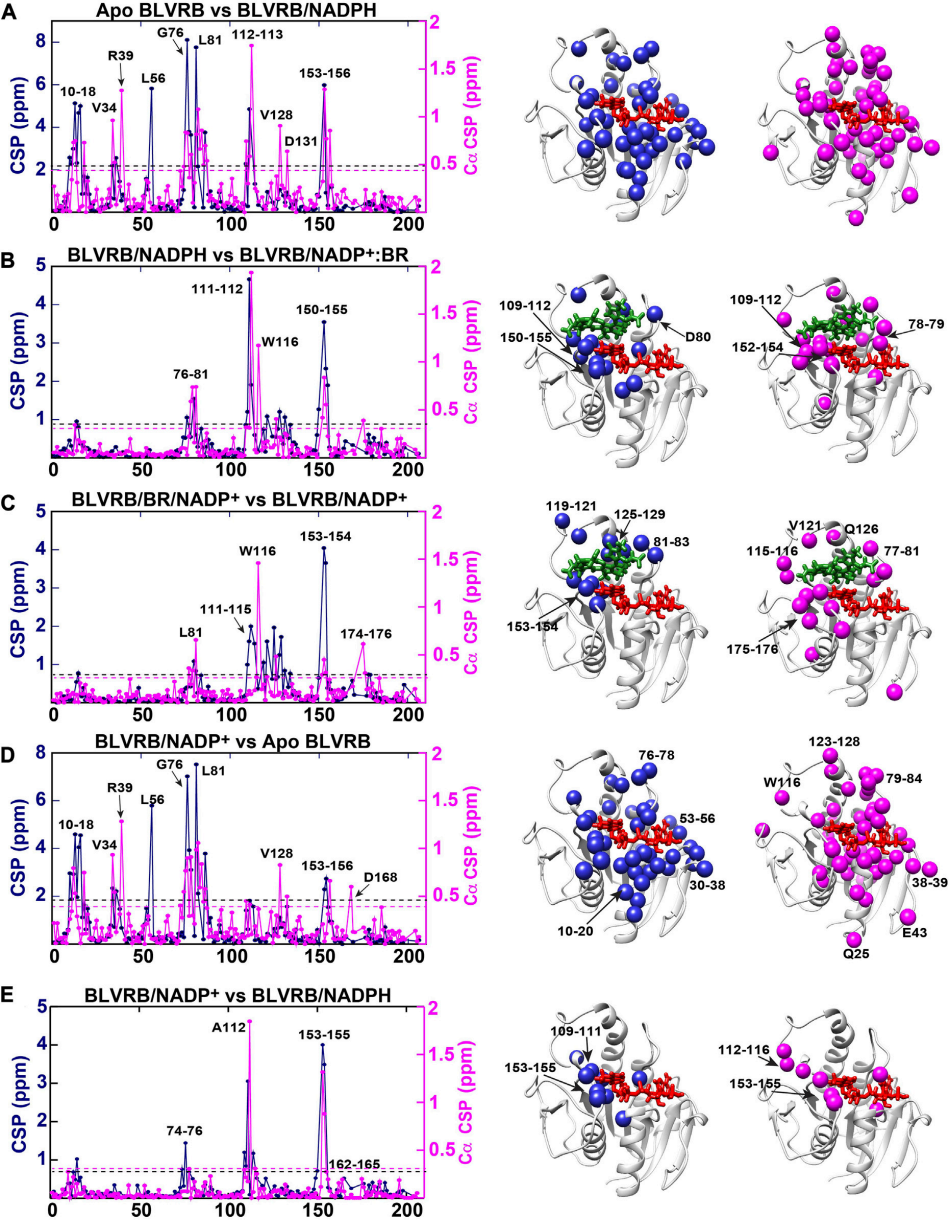
Amide and Cα CSPs between BLVRB catalytic intermediates. The absolute differences for amide CSPs (navy blue) and Cα CSPs (magenta) are shown (left). The same cutoffs were used to map CSPs in order to compare the number of residues affected. Specifically, while the average CSP plus one standard deviation is listed below quantified from these data, a hard cutoff of the lowest value was used for amide CSPs (center, navy blue balls that are larger than 0.78 ppm) and for Cα CSPs (right, magenta balls that are larger than 0.24 ppm). **(A)** Apo BLVRB *versus* BLVRB/NADPH with the average plus one standard deviation of 2.2 ppm for amide CSPs (black dashed line) and 0.49 ppm for Cα CSPs (magenta dashed line). **(B)** BLVRB/NADPH *versus* BLVRB/NADP+:BR with the average plus one standard deviation of 0.90 ppm for amide CSPs (black dashed line) and 0.31 ppm for Cα CSPs (magenta dashed line). **(C)** BLVRB/NADP+:BR *versus* BLVRB/NADP+ with the average plus one standard deviation of 0.83 ppm for amide CSPs (black dashed line) and 0.24 ppm for Cα CSPs (magenta dashed line). **(D)** BLVRB/NADP^+^ *versus* apo BLVRB with the average plus one standard deviation of 1.91 ppm for amide CSPs (black dashed line) and 0.42 ppm for Cα CSPs (magenta dashed line). **(E)** BLVRB/NADPH *versus* BLVRB/NADP^+^ with the average plus one standard deviation of 0.78 ppm for amide CSPs (dashed black line) and 0.26 ppm for Cα CSPs (magenta dashed line).

Backbone assignments facilitated the quantitative determination of both amide and Cα CSPs (Figure 3, left). We note that as we are unable to probe the catalytically active Michaelis-Menten complex of BLVRB/NADPH/biliverdin, differences between the prior and following step were calculated instead to identify changes between these intermediates (Figure 3B). In general, while both amide and Cα CSPs are sensitive markers to chemical environments, the backbone Cα chemical shifts are largely dictated by local secondary structure. For example, Cα chemical shifts are routinely compared to their shifts for the same residue within a random peptide (Hafsa and Wishart, 2014), which are called chemical shift propensities (defined as “ΔCα”). Propensities larger than that of a random peptide for the same residue indicate α-helical propensity (i.e., ΔCα>0) while Cα chemical shifts that are lower than that of a random peptide indicate β-strand propensity (ΔCα<0). For the case of BLVRB here, most of these propensities remain in the same direction (Supplementary Figure S1), suggesting that the secondary structure remains similar through the catalytic cycle. However, localized changes are observed for several residues. These include R78 that has no propensity in the apo state to largely helical propensity through the rest of the catalytic cycle. Interestingly, S112 adjacent to the coenzyme’s hydride is disordered only when bound to NADPH, suggesting localized flexibility that may be important for hydride transfer. Thus, while the secondary structure remains largely the same through the catalytic cycle, localized differences are observed throughout the enzyme that are further described below.

We mapped both the amide and Cα CSPs between intermediates to quantitatively identify regions exhibiting changes (Figure 3, middle and right). Several observations are immediately apparent for the CSPs monitored here between catalytic cycle intermediates. First, the same regions that exhibit amide CSPs overlap well with the regions that exhibit Cα CSPs. This indicates that changes in chemical environment through the catalytic cycle are likely concomitant with subtle changes to local secondary structure. Second, NADPH binding (Figure 3A) and NADP^+^ release (Figure 3D) induces the largest overall CSPs in both magnitude and number of residues. This is contrast to smaller changes in both magnitude and the number of residues induced by the substrate between intermediates (Figures 3B, C) and can be observed quantitatively by the reduced average changes for the substrate when compared to the coenzyme (Figures 3B, C *versus* Figures 3A, D). Third, many of the CSPs through the catalytic cycle are not immediately in contact with the coenzyme or bilirubin product. This indicates that long range allosteric changes are induced, which is consistent with our recent study that indicated mutation of BLVRB T164 within the C-terminal lobe induces measurable changes to the active site (Redzic et al., 2021). Finally, one of the most interesting findings here may be that the oxidation state of the coenzyme alone induces global CSPs (Figure 3E). Specifically, although the enzyme does not proceed directly between NADPH to NADP^+^ bound forms, a comparison between these two intermediates illustrates that the oxidative state of the coenzyme alone induces changes (Figure 3E). Interestingly, these CSPs are quantitatively on a similar level to the CSPs induced by bilirubin (Figures 3B, C). Moreover, an allosteric network of Cα CSPs includes residues 153–155 and residues 112–116 that reside in the substrate binding pocket. The fact that a single hydride and thus, the oxidative state of the nicotinamide group, can induce such largescale changes may have functional consequences for substrate binding. For example, subtle differences in coenzyme puckering or the dynamics of the coenzyme itself may differ through the catalytic cycle (Sundaresan et al., 2003; Tsybovsky et al., 2013). For BLVRB, many active site residues that straddle the entire coenzyme exhibit Cα CSPs between the oxidation states (Figure 3E), suggesting that subtle structural changes of to the coenzyme’s stereochemistry are indeed by coenzyme oxidation. Even more distal regions are also affected by the oxidative state that are simply below the threshold used here for mapping changes, such as residues within the C-terminal lobe that include residues 163–165. Thus, both local and allosteric changes can be monitored through the catalytic cycle with the coenzyme inducing the largest changes but the oxidative state of the coenzyme still modulating both local and allosteric changes.

## Mutations that “short-circuit” global hydride coupling alter catalytic function

**FIGURE 4:**
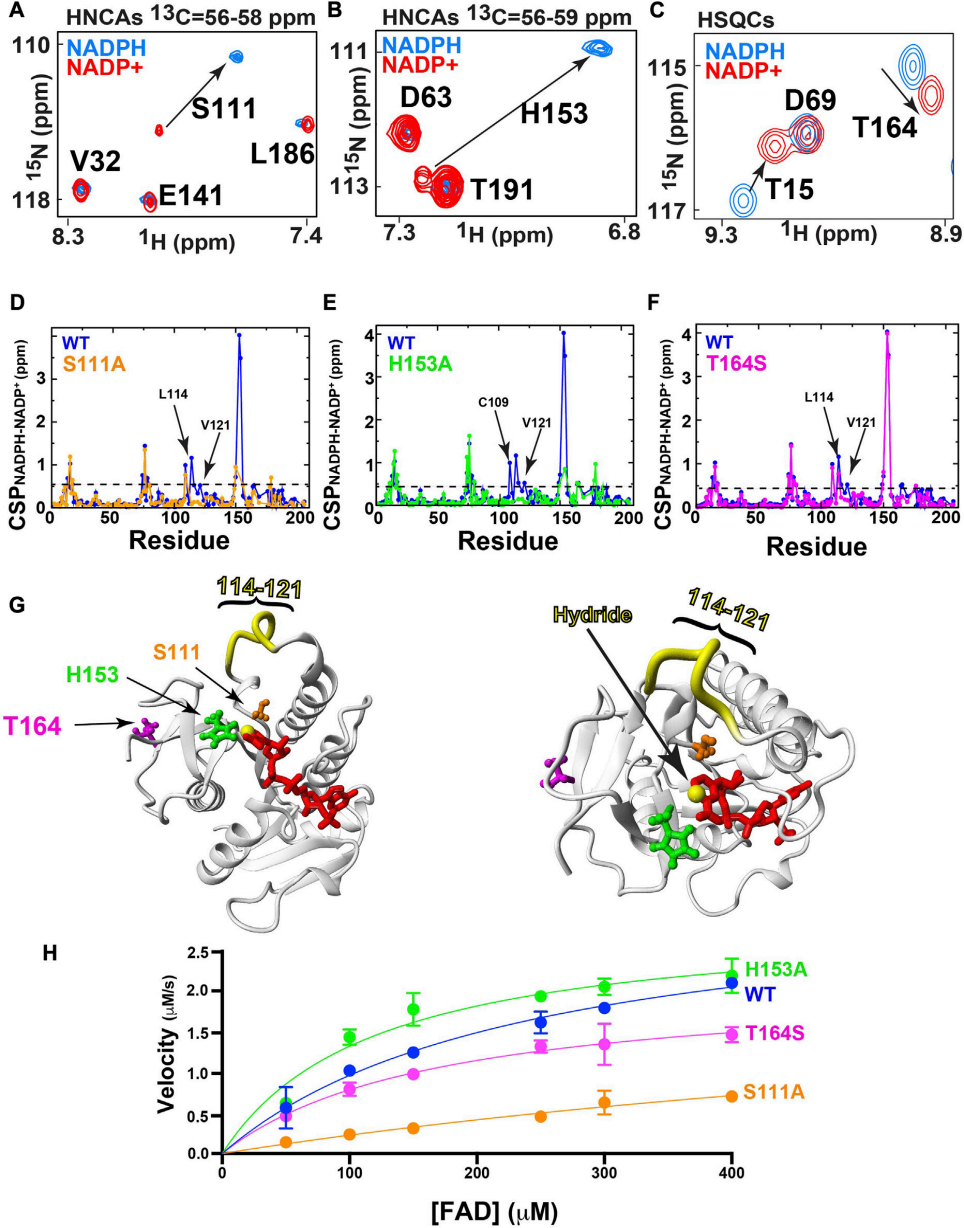
Mutagenic changes to hydride-mediated coupling and function. All ^15^N-HSQCs were collected at 20°C at 900 MHz and 3D-HNCAs collected at 20°C at 600 MHz A) The amide of S111A exhibits a significant CSP between reduced/oxidized forms of coenzyme. 3D HNCA planes are shown for S111 due to overlap in the ^15^N-HSQC spectra. **(B)** The amide of H153A exhibits a significant CSP between reduced/oxidized forms of coenzyme. 3D HNCA planes are shown for H153 due to overlap in the ^15^N-HSQC spectra. **(C)** The amide of T164S exhibits a significant CSP between reduced/oxidized forms of coenzyme, as shown in ^15^N-HSQC spectra. **(D)** Amide CSPs for NADPH/NADP^+^ of WT reported in Figure 3E (blue) compared to S111A (orange). The average CSP plus one standard deviation is show for S111A is 0.45 ppm (dashed line) **(E)** Amide CSPs for NADPH/NADP^+^ of WT reported in Figure 3E (blue) compared to H153A (green). The average CSP plus one standard deviation is show for H153A is 0.45 ppm (dashed line) **(F)** Amide CSPs for NADPH/NADP^+^ of WT reported in Figure 3E (blue) compared to T164S (magenta). The average CSP plus one standard deviation is show for T164S is 0.51 ppm (dashed line) **(G)** Two views of the positions of each mutation within the holo BLVRB structure and the region of residues 114–121 (yellow) that exhibits similar imparted changes from all three mutations. Mutations are colored the same as D-F and the C4N position that is reduced is shown (yellow sphere). **(H)** UV-kinetics turnover of FAD is shown for the WT and all three mutants. Specific Vmax values with KM values in parenthesis of each are as follows: WT is 3.3 ± 0.5 μM*s^-1^ (242 ± 70 μM), H153A is 2.9 ± 0.2 μM*s^-1^ (129 ± 40 μM), S111A is 2.5 ± 0.10 μM*s^-1^ (856 ± 300 μM), and T164S is 2.1 ± 0.01 μM*s^-1^ (171 ± 45 μM).

Considering that the oxidative state of the coenzyme alone, and thus a single hydride, modulates changes both within and distant to the active site (Figure 3E), we sought to further explore networks of allostery modulated by the oxidation state of the coenzyme. To further probe such coupling, we screened several BLVRB mutations both within and distal to the BLVRB active site using CSPs. Specifically, we selected three residues that incurred large CSPs between their bound reduced and oxidized forms of the coenzyme, which included S111 (Figure 4A), H153 (Figure 4B), and T164 (Figure 4C). These sites were also chosen as both S111 and H153 are immediately adjacent to the coenzyme’s hydride, while previous studies have indicated that T164 is allosterically coupled to the active site (Redzic et al., 2021). As mutation of T164 to a serine found in other mammalian homologues induces larger functional effects that a random mutation to an alanine (Redzic et al., 2021), we chose to explore T164S. Thus, S111A, H153A, and T164S were used to identify the effects to global hydride-coupled networks.

Amide CSPs between NADPH and NADP^+^ bound forms of BLVRB wild type (WT) calculated from ^15^N-HSQC spectra were compared to the respective differences for S111A (Figure 4D), H153A (Figure 4E), and T164S (Figure 4F). We note that the amides and several residues immediately adjacent to the mutated active site of S111A and H153A were not identified either due to overlap or severe line-broadening. Some local changes are observed to differ, such as H153A induced changes to neighboring residues C109. However, the most obvious impact of all three mutations was that hydride-mediated changes were similarly diminished for residues within the substrate binding loop of residues 114–121 (Figure 4G). In other words, whether active site residues are mutated, or a residue shown to be involved in allosteric coupling is mutated, their impacts on hydride-coupled networks are similar. We have previously referred to such diminished effects on either CSPs or the dynamics of networks of coupled residues as “short-circuited” (Holliday et al., 2017). Interestingly though, the catalytic effects of substrate turnover are different between these mutations using the FAD substrate (Figure 4H). FAD was used as no commercial sources of the biliverdin-beta isomer are available. Specifically, we compared turnover of these mutations to the BLVRB WT using the standard UV-kinetics assay under saturating conditions of NADPH while changing FAD (Cunningham et al., 2000; Duff et al., 2020). While we have previously presented similar catalytic data (Redzic et al., 2021), here we performed this assay differently in order to sample higher concentrations of FAD (described in Materials and Methods). Reversal of this UV-kinetics assay to vary NADPH is not attainable considering the high concentrations needed to saturate FAD that saturates the UV detector (Paukovich et al., 2018). However, using this assay while varying FAD, all three mutations induced changes to turnover. Both S111A and T164S mutations lead to reductions in maximum velocity and weaker affinities (higher K_M_ values) while H153A has no effect on maximum velocity but increase affinity (lower K_M_ value). Considering that the global CSPs short-circuited by these three mutants are largely similar, it is interesting to speculate why their imparted catalytic changes differ. Although such differences could be due to an array of perturbations during the catalytic cycle, catalytic differences could also be influenced by the way the oxidized and reduced forms of the coenzyme may lie within the active site of each mutant. In general though, these catalytic differences relative to the WT enzyme indicate that disturbing residues involved in hydride-coupled networks broadly affect catalytic function.

### Standard relaxation rates reveal that the dynamic regions are largely similar through the catalytic cycle

Enzymes are not static but instead, are often inherently dynamic, which may be particularly important to allow for the conformational changes that ensue during catalytic function. Standard relaxation methods were therefore applied to BLVRB catalytic intermediates to determine how dynamics may change during the catalytic cycle. These include R1 relaxation rates that are sensitive to the ps-ns timescales and R2/R1ρ relaxation rates that are sensitive to the slower μs-ms timescales, which are both extracted from their time dependencies with examples and their fits shown here (Supplementary Figure S2).

**FIGURE 5:**
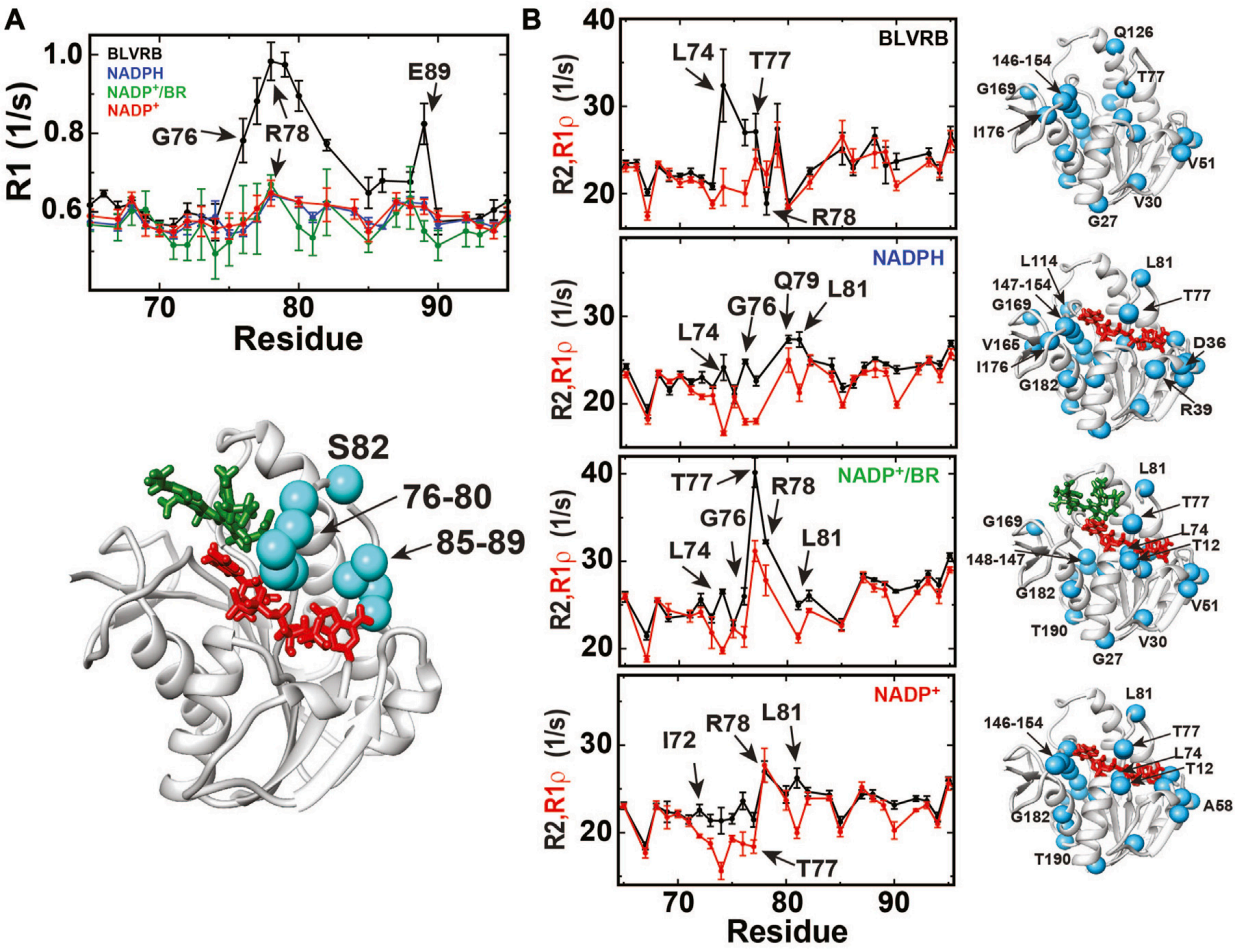
R1, R2, and R1ρ relaxation rates monitored for BLVRB catalytic intermediates. **(A)** Top: R1 relaxation rates of apo BLVRB (black), BLVRB bound to NADPH (blue), BLVRB bound to NADP^+^ and BR (green), and BLVRB bound to NADP^+^ (red). R1 relaxation rates for residues 60–100 are shown. Bottom: R1 relaxation rates within apo BLVRB greater than one standard deviation mapped onto a model of the ternary complex, which is quenched for all other intermediates. **(B)** R2 and R1ρ relaxation rates are shown for BLVRB catalytic intermediates with significant differences larger than their uncertainties mapped onto the model structure of the ternary complex for each intermediate (cyan spheres). R2 and R1ρ relaxation rates for residues 60–100 are shown. All data were collected at 20°C at 900 MHz with all data shown in Supplementary Figure S3, 4.

R1 relaxation data indicate that the disordered R78-loop in apo BLVRB remains largely ordered upon coenzyme binding through the catalytic cycle (Figure 5A; Supplementary Figure S3). This can be structurally explained by the likely preservation of the hydrogen bond between the R78 side chain and R12 CO observed within the X-ray crystal structure of BLVRB bound to NADP^+^ (Pereira et al., 2001). It should be noted that R1 relaxation rates are strictly a weighted average and thus, these findings do not exclude the likely possibility that this loop must further open to facilitate NADPH binding and NADP^+^ release. However, such R1 relaxation rates do imply that once the R78-loop is closed upon NADPH binding that it stays relatively ordered through the catalytic cycle.

Chemical exchange that contributes to R2 relaxation in the μs-ms timescale are suppressed by the imparted refocusing field within R1ρ relaxation and therefore identify slower motions within BLVRB that persist through the catalytic cycle (Supplementary Figure S4). Specifically, amides that exhibit differences between R2 and R1ρ greater than their respective uncertainties indicate that many of the same residues exhibit exchange that include residues within the R78-loop (Figure 5B) and within residues 164–177 (Supplementary Figure S4). This means that many of the same regions of BLVRB are moving within the μs-ms timescale during multiple steps of the catalytic cycle. However, the fact that such motions persist within multiple intermediates within the catalytic cycle does not necessarily imply that their motions are equivalent. Thus, we subjected BLVRB catalytic intermediates to R2-CPMG dispersion experiments and did so at multiple temperatures, as described below.

### R2-CPMG dispersion experiments quantify differential dynamics during the BLVRB catalytic cycle

Based on R2/R1ρ relaxation rate comparisons described above that revealed similar regions exhibit μs-ms timescale motions within different catalytic intermediates, we sought to understand whether the underlying motions differ between intermediates. However, our previously published R2-CPMG studies that focused solely on comparisons between the apo and NADP^+^ bound forms at 20°C revealed several challenges that required alternative strategies (Paukovich et al., 2018). First, exchange contributions with NADP^+^ bound were very small at 20°C. Such small contributions preclude data collection at multiple static fields that are required to extract the associated biophysical parameters of motions (Kovrigin et al., 2006). Second, our recent studies indicate that much of the exchange dynamics within BLVRB are highly localized but nonetheless partially coupled throughout the enzyme (Redzic et al., 2021). This means that R2-CPMG fits to a two-site exchange are in general confounded by contributions from numerous processes and are therefore an over-simplification. To partially address these challenges, R2-CPMG dispersions were collected at lower temperatures of 0^o^C, 5^o^C, and 10^o^C at 900 MHz where exchange was larger in some intermediates. To estimate the underlying physical parameters that define μs-ms timescale motions within these BLVRB intermediates, amides that exhibited similar responses within each intermediate were simultaneously grouped and fit at multiple temperatures using the Arrhenius dependence (Millet et al., 2000; Vallurupalli and Kay, 2006).

**FIGURE 6:**
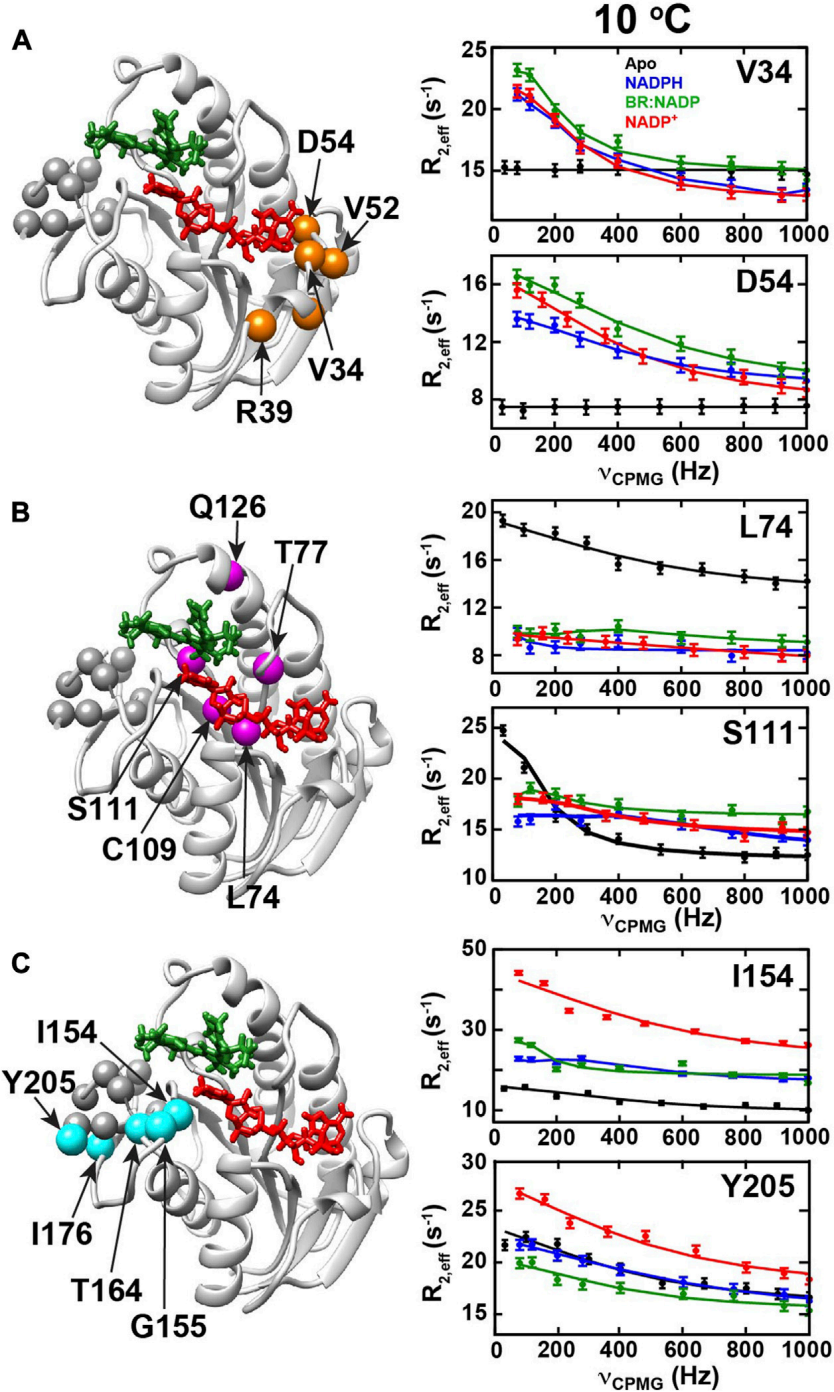
R2-CPMG dispersion profiles for BLVRB catalytic intermediates. R2-CPMG dispersions are shown for three groups of amides that exhibit similar trends for apo BLVRB (black), BLVRB bound to NADPH (blue), BLVRB bound to NADP+ and BR (green), and BLVRB bound to NADP^+^ (red). Intensity-based rates are shown as a function of the imparted CPMG field, ν_cpmg_. **(A)** Group I residues are mapped onto the ternary complex (orange spheres) that include V34, D36, R39, V52, and D54 with R2-CPMG dispersions of V34 and D54 amides shown at 10°C. **(B)** Group II residues are mapped onto the ternary complex (magenta spheres) that include L74, T77, C109, H111, and Q126 with L74 and S111 amide R2-CPMG dispersions shown at 10°C. **(C)** Group III residues that include I154, G155, T164, I176, Y205 are mapped onto the ternary complex with I154 and Y205 amide dispersions shown at 10°C. All data were collected at 900 MHz and all R2-CPMG dispersions are shown with their fits in Supplementary Figure S5.

R2-CPMG dispersions collected here reveal that their profiles change for many sites through the catalytic cycle that indicates the associated dynamics also differ (Supplementary Figure S6). Three groups exhibiting similar changes to their R2-CPMG dispersions were identified, which are referred to herein as “Group I”, “Group II”, and “Group III” (Figure 6). These three groups were then separately fit using Graphical User-friendly Analysis of Relaxation Dispersion Data (GUARDD) (Kleckner and Foster, 2012). The extracted rates of exchange and populations are tabulated here within the three groups at the three temperatures (Table 2). Specifically, amides within Group I exhibit no exchange contributions within apo BLVRB but exhibit similar R2-CPMG dispersions once coenzyme and product are bound (Figure 6A). Several Group I residues make direct contact to the adenine moiety of the adenosine. The opposite is true for amides within Group II (Figure 6B), which exhibit large exchange in apo BLVRB but have this exchange largely quenched within the other intermediates with little to no R2-CPMG dispersion. However, some of these residues do still exhibit small exchange contributions that increase with lower temperatures with both the reduced and oxidized coenzyme that could be fit to relatively fast exchange rates (Table 2). While this indicates that at least some movements within Group II simply shift to faster rates of exchange (and thus, smaller exchange contributions), there is no observable exchange in the presence of the BR product at multiple temperatures (Supplementary Figure S5). Group II residues are scattered closer to the nicotinamide and substrate binding site. Finally, Group III residues exhibit detectable exchange throughout the catalytic cycle with subtle differences between each intermediate (Figure 5C). R2-CPMG dispersion is even different between the oxidized and reduced form of the coenzyme, suggesting that, like CSPs, the oxidative state of the coenzyme is allosterically coupled to other regions of BLVRB. Interestingly, the associated rates of motions of Group III exhibit the least relative changes as a function of temperature (Table 2). These residues are located within the C-terminal lobe of BLVRB, which also includes residues within 166–175 that remain unobservable (except for G169) within all intermediates. Thus, although many similar regions are moving within multiple intermediates, the specific motions themselves do differ through the catalytic cycle.

**TABLE 2:** Exchange rates and populations determined for three separate groups. Residues within each group were globally fit using 0°C, 5°C, and 10°C data except for apo BLVRB that also included 20°C. Data were fit using GUARDD (Kleckner and Foster, 2012).

	Group I	Group II	Group III
Apo (20°C)	-	1088 ± 180 Hz (97.8 ± 0.2%)	3797 ± 800 Hz (98.8 ± 0.1%)
Apo (10°C)	-	457 ± 03 Hz (96.9 ± 0.4%)	1846 ± 307 Hz (98.4 ± 0.1%)
Apo (5°C)	-	290 ± 93 Hz (96.3 ± 0.8%)	1263 ± 222 Hz (98.1 ± 0.2%)
Apo (0°C)	-	181 ± 66 (95.5 ± 1.5%)	850 ± 178 Hz (97.8± 0.3%)
NADPH (10°C)	1206 ± 175 Hz (98.7 ± 0.1%)	1937 ± 480 Hz (96.4 ± 3.1%)	4480 ± 922Hz (95.6 ± 3.3%)
NADPH (5°C)	835 ± 114 Hz (98.3 ± 0.2%)	988 ± 162 Hz (93.4 ± 5.4%)	4024 ± 890 Hz (95.2 ± 3.6%)
NADPH (0°C)	572 ± 105 Hz (97.7 ± 0.4%)	505 ± 111 Hz (87.9 ± 9.0%)	3604 ± 867 Hz (94.7 ± 3.9%)
NADP+/BR (10°C)	1590 ± 111 Hz (98.6 ± 0.1%)	-	3000 ± 1350 Hz (98.6 ± 3.7%)
NADP+/BR (5°C)	1252 ± 90 Hz (98.6 ± 0.1%)	-	2085 ± 1184 Hz (98.1 ± 4.7%)
NADP+/BR (0°C)	978 ± 107 Hz (98.5 ± 1%)	-	1430 ± 1035 Hz (97.4 ± 5.9%)
NADP+(10°C)	1850 ± 77 Hz (98.6 ± 0.1%)	3884 ± 450 Hz (99.3 ± 0.1%)	4117 ± 1100 Hz (97.4 ± 0.6%)
NADP+(5°C)	1000 ± 38 Hz (98.3 ± 0.1%)	2685 ± 380 Hz (99.0 ± 0.2%)	3820 ± 1160 Hz (97.2 ± 0.7%)
NADP+(0°C)	530 ± 33 Hz (98.1 ± 0.1%)	1833 ± 340 Hz (98.6 ± 0.2%)	3536 ± 1360 Hz (96.9 ± 0.8%)

To help understand the physical basis of the dynamically sampled conformations monitored by R2-CPMG dispersions, we compared the calculated chemical shift changes from the fits (Δω_cpmg_) to the experimental CSPs measured between all possible BLVRB intermediates (Supplementary Figure S6). Specifically, if sampled populations are identical to either the previous conformation within the catalytic cycle (backward sampling) or the next sampled conformer (forward sampling), there should be a correlation between the calculated and experimental chemical shifts. While such comparisons are often complicated by the fact that the sampled chemical environment may differ in the presence of the bound species, we do observe at least several weak correlations for some intermediates. Specifically, several amides within apo BLVRB appear to dynamically sample similar CSPs to their holo forms (Supplementary Figure S6, top). Interestingly, only I176 and Y205 that are both within the C-terminal lobe of BLVRB do not sample conformations with similar CSPs. Beyond apo BLVRB, about half the measured dynamics within NADP^+^-bound BLVRB dynamically sample similar CSPs to the apo form (Supplementary Figure S6, bottom). Our inability to monitor the Michaelis-Menten complex prevents us from specifically comparing many of the other dynamically sampled conformations (Supplementary Figure S6, middle). Nonetheless, the remaining comparisons do not indicate that dynamics within other intermediates sample similar conformations to those defined by their CSPs. Thus, apo and holo BLVRBs partially sample each other’s conformations, which is supported by the apo BLVRB structure solved here that is similar to that of holo BLVRB.

## Discussion

### Structural changes between BLVRB catalytic intermediates

This study identified regions of BLVRB that incur conformational changes during different steps of its catalytic cycle. We began by successfully solving X-ray crystal structures of the apo form of BLVRB for the first time at both cryogenic and ambient temperatures, which had previously eluded crystallization attempts. The comparison between the holo BLVRB structure and the apo BLVRB structures revealed that several active site residues undergo conformational changes to accommodate the coenzyme, including R35, R39, and R78. One important difference between the apo BLVRB structures was that the apo-cryo structure exhibited a defined density of the R78-loop that would block coenzyme entry and release, while this region was disordered in the apo-RT structure that would facilitate such an interaction. Such comparisons highlight the potential misinterpretation of structures determined by cryogenic crystallography without complementary ambient temperature experiments. Importantly, even in the apo-RT BLVRB structure, the hydrogen bond between R78 and the T12 CO observed within the NADP^+^-bound holo BLVRB structure was not formed. This is also consistent with the high flexibility of the R78-loop in the apo state (Paukovich et al., 2018). Additional local differences within the crystals between the apo and holo BLVRB structures were observed distant to the active site, illustrating allosteric rearrangements throughout the enzyme.

NMR solution studies that focused on amide and Cα CSPs revealed environmental and secondary structural changes between BLVRB catalytic intermediates both within the active site and distal regions, consistent with allosterically coupled changes. The largest changes were found to occur between BLVRB intermediates upon coenzyme binding, whether reduced or oxidized, compared to more localized changes due to product interactions. This suggests that global rearrangements are induced to a larger degree by coenzyme interactions relative to the product. Interestingly, the oxidative state of the coenzyme, meaning the differences between NADPH-bound and NADP^+^-bound, exhibited CSPs on a similar scale to those induced by the product. While such changes within the active site could reflect slight differences in the stereochemistry of the coenzyme that are altered by the oxidation state, as has been observed before (Sundaresan et al., 2003; Tsybovsky et al., 2013), distal residues are nonetheless affected. Thus, the oxidative state of the coenzyme induces allosterically coupled changes. Mutations that alter this coupling to the oxidative state, i.e., that alter hydride-mediated coupling, also exhibit catalytic differences. We have therefore shown that allosteric coupling to the oxidation state of a reductase’s coenzyme is important for function.

### Dynamic changes between BLVRB catalytic intermediates

We utilized NMR relaxation rate measurements to reveal the changes that occur within BLVRB during different steps of the catalytic cycle. For the ps-ns timescale, the inherently dynamic R78-loop in the apo state remains largely ordered for the other catalytic intermediates. This suggests that once the coenzyme is in place, the R78-loop is stabilized by the hydrogen bond between the side chain of R78 and the T12 CO observed within the NADP^+^-bound X-ray crystal structure (Pereira et al., 2001). For slower μs-ms timescales, comparisons of R2 relaxation with R1ρ relaxation were initially used to identify sites of dynamic exchange within BLVRB catalytic intermediates. These comparisons revealed that exchange contributions were quenched within similar regions of BLVRB through the catalytic cycle, indicating that the associated dynamic exchange on the μs-ms timescale is relegated to similar regions. By applying R2-CPMG dispersion experiments to further quantify the dynamic exchange within BLVRB at multiple temperatures, we identified groups of residues that exhibit similar patterns, or responses, through the catalytic cycle that were structurally localized to different regions. Thus, despite similar regions moving on the μs-ms timescale within different intermediates, the dynamic processes themselves differ through the catalytic cycle.

Using R2-CPMG dispersions, we identified three groups of residues that exhibit similar μs-ms motions within BLVRB intermediates, which included both the active site as well as allosterically coupled residues. Two of these groups were within the active site (Group I and II) and appeared to undergo a dynamic “switch”. Specifically, Group I exhibited no R2-CPMG dispersion in apo BLVRB but did exhibit dispersion throughout the rest of the catalytic cycle and vice-versa for Group II that was inherently dynamic within apo BLVRB but largely quenched with coenzyme and substrate. In contrast, the final group (Group III) was both proximal to the active site but extended well within the C-terminal lobe. The identification of coupled movements within this C-terminal lobe are consistent with our previous studies that have identified one particular site, T164, which modulates coenzyme affinity allosterically (Redzic et al., 2021). Specifically, the movements within this C-terminal region are drastically altered by mutation of T164 and this directly impacts coenzyme affinity. Just as mutation of T164 has previously been shown to alter active site dynamics, here we showed that even the oxidative state of the coenzyme affects the dynamics within these allosterically coupled sites. Specifically, R2-CPMG dispersions are not identical between bound NADPH and NADP^+^ for these residues within Group III. Interestingly, the rate of exchange within this group extracted from group fitting of R2-CPMG dispersions here also revealed the least change with temperature. This observation that BLVRB retains the underlying movements of this C-terminal lobe to similar rates of motions can be compared to other enzymes that have been observed to limit temperature dependencies within catalytically important regions (Schlegel et al., 2009a). For example, a limited temperature dependence may be an evolutionary feature to maintain catalytic efficiencies. The importance of this C-terminal region comprising Group III residues is also consistent with comparisons to other SDR family members. Specifically, the C-terminal lobe of BLVRB is the distinguishing feature among SDR family members that dictates substrate interactions (Kavanagh et al., 2008).

Finally, it is important to note the dynamic differences of BLVRB to another well-known reductase, DHFR. Unlike concerted motions identified for DHFR during its catalytic cycle that enable sampling of either the previous or following catalytic step (Boehr et al., 2006a; Boehr et al., 2006b), we observed only weak evidence of such concerted movements within BLVRB. Specifically, only in the case of either apo BLVRB or holo BLVRB bound to NADP^+^ did we observed a subset of CSPs that were similar to the calculated chemical shift differences derived from R2-CPMG dispersion. While this is at least consistent with our X-ray crystal structures of apo BLVRB that is indeed similar to bound NADP^+^, the dynamics within the remaining BLVRB intermediates appear not to sample previous or following conformations. Moreover, unlike DHFR where dynamics have been associated with the rate-limiting step of product release (Boehr et al., 2008), BLVRB turnover is much slower than any of the independent steps that include hydride transfer (Duff et al., 2020). This suggests that the dynamic relationship of BLVRB to function may be a collection of dynamic events that influence catalysis rather than a single movement dictating catalysis. Nonetheless, this study revealed dynamic differences within each intermediate through the catalytic cycle within both the active site and allosterically coupled sites.

## Methods

### Protein expression and purification

Expression and purification of recombinant human BLVRB (UniProt P30043) has previously been described (Paukovich et al., 2018). Briefly, BLVRB cloned after an N-terminal 6xHis-tag and thrombin cleavage site was expressed in pET21 was expressed in BL21/DE3 cells at 37^o^C and induced at 0.6 ODs (600 nM) with isopropyl b-D-1-thiogalactopyranoside (IPTG). BLVRB mutations were produced by a nested PCR protocol whereby the initial PCR produced a partial fragment with the mutation that utilized this fragment as a primer for the full-length PCR fragment with insertion into NdeI-cleaved pET22. Hi-Fi PCR amplification (NEB) was used for all PCR reactions and In-Fusion (Takara) was used for vector insertion. Unlabeled proteins were expressed in luria broth (LB) for 3 h and labeled proteins were expressed in M9 minimal media for 5 h ^2^H,^15^N,^13^C-labeling for backbone assignments was grown in 99.9% D_2_O M9 minimal media supplemented with ^15^N ammonium chloride and ^13^C glucose facilitated further assignments of residues initially too line broadened. ^2^H,^15^N-labeling for R2-CPMG dispersion experiments was grown in 99.9% D_2_O M9 minimal media supplemented with ^15^N ammonium chloride. BLVRB proteins were all purified denatured via Ni-affinity (Sigma), refolded through dialysis, and further purified via Superose-75 size-exclusion-chromatography (Cytiva) as previously described (Paukovich et al., 2018; Duff et al., 2020; Redzic et al., 2021).

Human glucose-6-phosphate dehydrogenase (G6PD, UniProt P11413) that was used to recycle NADPH, described further below, was also cloned into pET21 for expression at 25^o^C and induced at 0.6 ODs (600 nM) with IPTG for 5 h. Soluble protein was purified via Ni-affinity and fractions concentrated for further purification of the tetramer via Superose-200 size-exclusion-chromatography (Cytiva).

### Crystallization and structure solution

Crystals were grown by vapor diffusion where protein/mother liquor drops were equilibrated over a 0.25–0.5 M NaCl solution at 25 °C. 3 μL of human BLVRB at 18 mg/mL were mixed with 3 µL of crystallization solution consisting of 0.1M MES pH 6.5% and 1%–4% PEG 20000 (Duff et al., 2020). Initial crystals were harvested for seeds of which 0.5 µL were added to fresh drops after 1 day of equilibration. Crystals were cryoprotected in the crystallization solution with the addition of 10% PEG 400. Crystals were either immersed into liquid nitrogen for cryogenic data collection or wrapped in NVH oil for ambient temperature collection. Data was collected at CHESS ID7B2 beamline using CRLs. Data was index and scaled in DIALS (Winter et al., 2018) and merged using AIMLESS (Evans and Murshudov, 2013). BLVRB structures were solved using MOLREP in the CCP4 suite with PDB accession 1HDO as a search model (Bailey, 1994; Pereira et al., 2001; Vagin and Teplyakov, 2010). Real-space refinement, model building, heteroatom and water addition was completed in COOT (Emsley and Cowtan, 2004). Refinement was completed using Phenix. refine using automatic TLS restraints (Afonine et al., 2012). Validation was completed using Molprobity server (http://molprobity.biochem.duke.edu/index.php).

### NMR sample preparation, spectroscopy, and data analysis

All BLVRB samples were prepared in 50 mM bis-tris, pH 6.5, 50 mM NaCl, 1 mM DTT at 500 μM protein with 5% D_2_O. Holo BLVRB with NADP^+^ (oxidized coenzyme) comprised 2 mM coenzyme while samples with the coenzyme product also comprised 700 μM bilirubin. For holo BLVRB with NADPH, the recycling system previously exploited was used to extend the life-time of the sample due to the short half-life of the reduced form of this coenzyme (Boehr et al., 2006b). Specifically, 2 mM NADPH was used with 10 μM G6PD and 10 mM G6P. No interaction was observed for G6P with BLVRB, as assessed by ^15^N-HSQC spectra. Backbone assignments using a ^2^H,^15^N,^13^C-labeled enzymes were collected on a Bruker 600 spectrometer equipped with a cryo-probe at 20°C for all intermediates. These included an HNCA, HN(co)CA, HNCACB, and a HN(co)CACB. As the Cα resonances are readily obtained from both the HNCA and HN(co)CA, both the HNCACB and CBCA(co)NH were optimized for CB coupling, specifically by setting the delay for CC coupling to 6 m. All 3D assignment spectra utilized non-uniform sampling (NUS) reconstructed to 72 and 96 increments in the nitrogen and carbon dimensions, respectively (Hyberts et al., 2012). Backbone assignments for BLVRB in the presence of NADPH and in the presence of NADP^+^ and bilirubin have been deposited into the Biological Magnetic Resonance Data Bank (BMRB) with accession numbers 51658 and 51657, respectively, and BLVRB alone and in the presence of NADP^+^ have previously been deposited (Paukovich et al., 2018).

All relaxation experiments were conducted on the Rocky Mountain Varian 900 spectrometer equipped with a cryo-probe at 20^o^C with R1, R2, and R1ρ relaxation experiments utilizing a standard BioPack sequence and R2-CPMG dispersions were collected at 0^o^C, 5^o^C, and 10^o^C with TROSY selection as previously described (Schlegel et al., 2009b; Holliday et al., 2017). Standard R1, R2, and R1ρ relaxation rates were determined from fits in CCPNmr (Vranken et al., 2005) and R2-CPMG dispersions were fit to extract the associated exchange rates and populations using GUARDD (Kleckner and Foster, 2012).

Cα CSPs atoms were calculated by taking absolute differences between each intermediate while amide CSPs were calculated by the square root of the sum of squares for both nitrogen and protein shifts between intermediates. For hydride-mediated coupling experiments, BLVRB WT, S111A, H153A, and T164S were compared between reduced and oxidized coenzyme. Specifically, either 2 mM NADP^+^ or NADPH with the above-described recycling system were used and amide CSPs calculated accordingly.

### UV-kinetic assays

A Varian Cary 50 UV-Vis Spectrophotomer was used with a 1 mm cell and 300 μL total volumes to observe the diminishment in NADPH at 340 nm, as previously described (Cunningham et al., 2000). NADPH concentrations were kept constant at 300 μM, BLVRB and mutants were kept constant at 10 μM, and the FAD substrate was varied as indicated. Each velocity was determined from an average of three kinetic runs with initial velocities calculated for the first 60 s and fit to the Michaelis-Menton equation using GraphPad Prism version 4.0 (GraphPad Software Inc., San Diego, CA).

## Data Availability

The original contributions presented in the study are included in the article/Supplementary Material, further inquiries can be directed to the corresponding author.

## Supplementary material

Supplementary Figure S1 Cα chemical shift propensities for BLVRB catalytic intermediates. Backbone Cα chemical shift propensities (∆Cα) were calculated based on their differences to the same residue within a random coil (Hafsa and Wishart, 2014). These are shown for apo BLVRB (black), BLVRB/NADPH (blue), BLVRB/NADP^+^/bilirubin (green), and BLVRB/NADP^+^ (red). Arrows delineate residues that have very different propensities within at least one intermediate compared to others.

Supplementary Figure S2 Example data and fits for individual relaxation rates. Standard R1 and R2 relaxation rates and their fits using a single mono exponential for G102 are shown. All data were collected at 900 MHz at 20°C.

Supplementary Figure S3 R1 relaxation rates of BLVRB catalytic intermediates. All data were collected at 20°C at 900 MHz. **(A)** R1 relaxation rates for apo BLVRB (left) and rates larger than the average (0.663 s^−1^) plus ½ standard deviation (0.722 s^−1^) mapped onto the BLVRB structure (right). **(B)** R1 relaxation rates for holo BLVRB bound to NADPH (left) and rates larger than the average (0.612 s^−1^) plus ½ standard deviation (0.656 s^−1^) mapped onto the BLVRB structure (right). **(C)** R1 relaxation rates for holo BLVRB bound to NADPH and bilirubin (left) and rates larger than the average (0.585 s^−1^) plus ½ standard deviation (0.637 s^−1^) mapped onto the BLVRB structure (right). **(D)** R1 relaxation rates for holo BLVRB bound to NADP^+^ (left) and rates larger than the average (0.615 s^−1^) plus ½ standard deviation (0.657 s^−1^) mapped onto the BLVRB structure (right).

Supplementary Figure S4 R2 and R1ρ relaxation rates of BLVRB catalytic intermediates. R2 relaxation rates (black) and R1ρ relaxation rates (red) are shown. All data were collected at 20°C at 900 MHz. **(A)** R2/R1ρ relaxation rates for apo BLVRB (left) with residues exhibiting greater than the average differences (2.913 s^−1^) plus 1/4^th^ standard deviation (4.048 s^−1^) plotted onto the BLVRB structure (right). **(B)** R2/R1ρ relaxation rates for holo BLVRB bound to NADPH (left) with residues exhibiting greater than the average differences (2.790 s^−1^) plus 1/4^th^ standard deviation (3.910 s^−1^) plotted onto the BLVRB structure (right). **(C)** R2/R1ρ relaxation rates for holo BLVRB bound to NADPH and bilirubin (left) with residues exhibiting greater than the average differences (1.990 s^−1^) plus 1/4^th^ standard deviation (2.542 s^−1^) plotted onto the BLVRB structure (right). **(D)** R2/R1ρ relaxation rates for holo BLVRB bound to NADP^+^ (left) with residues exhibiting greater than the average differences (3.590 s^−1^) plus 1/4^th^ standard deviation (5.410 s^−1^) plotted onto the BLVRB structure (right).

Supplementary Figure S5 R2-CPMG dispersions for BLVRB catalytic intermediates at multiple temperatures. R2-CPMG dispersion profiles are shown for all residues exhibiting exchange in one BLVRB intermediate greater than 1.0 s^−1^. Data were fit using GUARDD (Kleckner and Foster, 2012) and the indicated temperatures collected for each set of dispersions are shown on the top. All data were collected at 900 MHz.

Supplementary Figure S6 Fit chemical shift differences compared to measured CSPs. R2-CPMG dispersions were used to calculate the associated chemical shift differences to a minor population of each individual group defined in Figure 5 using GUARDD (∆ωcpmg) and plotted against the measured CSPs between intermediates. The catalytic cycle is shown in the middle with the CSP comparisons to the previous intermediates (“Backward sampling”) and comparisons to the following intermediates (“Forward sampling”) shown to the left and right of the catalytic cycle, respectively. All temperatures of 0 C, 5 C, and 10 C.

## References

[B1] Afonine P. V. Grosse-Kunstleve R. W. Echols N. Headd J. J. Moriarty N. W. Mustyakimov M. (2012). Towards automated crystallographic structure refinement with phenix.refine. Acta Crystallogr. Sect. D-Structural Biol. 68, 352–367. 10.1107/s0907444912001308 PMC3322595 22505256

[B2] Bailey S. (1994). The CCP4 suite - programs for protein crystallography. Acta Crystallogr. Sect. D-Biological Crystallogr. 50, 760–763. 10.1107/s0907444994003112 15299374

[B3] Boehr D. D. Dyson H. J. Wright P. E. (2006a). An NMR perspective on enzyme dynamics. Chem. Rev. 106, 3055–3079. 10.1021/cr050312q 16895318

[B4] Boehr D. D. Dyson H. J. Wright P. E. (2008). Conformational relaxation following hydride transfer plays a limiting role in dihydrofolate reductase catalysis. Biochemistry 47, 9227–9233. 10.1021/bi801102e 18690714 PMC2562322

[B5] Boehr D. D. McElheny D. Dyson H. J. Wright P. E. (2006b). The dynamic energy landscape of dihydrofolate reductase catalysis. Science 313, 1638–1642. 10.1126/science.1130258 16973882

[B6] Cui D. S. Lipchock J. M. Brookner D. Loria J. P. (2019). Uncovering the molecular interactions in the catalytic loop that modulate the conformational dynamics in protein tyrosine phosphatase 1B. J. Am. Chem. Soc. 141, 12634–12647. 10.1021/jacs.9b04470 31339043 PMC8259405

[B7] Cunningham O. Gore M. G. Mantle T. J. (2000). Initial-rate kinetics of the flavin reductase reaction catalysed by human biliverdin-IXβ reductase (BVR-B). Biochem. J. 345, 393–399. 10.1042/bj3450393 10620517 PMC1220769

[B8] Duff M. R. Redzic J. S. Ryan L. P. Paukovich N. Zhao R. Nix J. C. (2020). Structure, dynamics, and function of the evolutionarily changing biliverdin reductase B family. J. Biochem. 168 (2), 191–202. D - 0376600, 1756-2651. 10.1093/jb/mvaa039 32246827

[B9] Eisenmesser E. Capodagli G. C. Armstrong G. S. Holliday M. Isern N. G. Zhang F. (2015). Inherent dynamics within the Crimean-Congo Hemorrhagic fever virus protease are localized to the same region as substrate interactions. Protein Sci. 24, 651–660. 10.1002/pro.2637 25564798 PMC4420516

[B10] Emsley P. Cowtan K. (2004). Coot: model-building tools for molecular graphics. Acta Crystallogr. Sect. D-Biological Crystallogr. 60, 2126–2132. 10.1107/S0907444904019158 15572765

[B11] Evans P. R. Murshudov G. N. (2013). How good are my data and what is the resolution? Acta Crystallogr. Sect. D-Biological Crystallogr. 69, 1204–1214. 10.1107/s0907444913000061 PMC3689523 23793146

[B12] Hafsa N. E. Wishart D. S. (2014). CSI 2.0: A significantly improved version of the chemical shift index. J. Biomol. Nmr 60, 131–146. 10.1007/s10858-014-9863-x 25273503

[B13] Holliday M. Camilloni C. Armstrong G. S. Vendruscolo C. Eisenmesser E. Z. (2017). Networks of dynamic allostery regulate enzyme function. Structure 25, 276–286. 10.1016/j.str.2016.12.003 28089447 PMC5336394

[B14] Huan L. Bao C. Y. Chen D. Li Y. Lian J. W. Ding J. (2016). MicroRNA-127-5p targets the biliverdin reductase B/nuclear factor-B pathway to suppress cell growth in hepatocellular carcinoma cells. Cancer Sci. 107, 258–266. 10.1111/cas.12869 26708147 PMC4814244

[B15] Hyberts S. G. Milbradt A. G. Wagner A. B. Arthanari H. Wagner G. (2012). Application of iterative soft thresholding for fast reconstruction of NMR data non-uniformly sampled with multidimensional Poisson Gap scheduling. J. Biomol. Nmr 52, 315–327. 10.1007/s10858-012-9611-z 22331404 PMC3321367

[B16] Iverson D. B. Xiao Y. Jones D. N. Eisenmesser E. Z. Ahn N. G. (2020). Activation loop dynamics are coupled to core motions in extracellular signal-regulated kinase-2. Biochemistry 59, 2698–2706. 10.1021/acs.biochem.0c00485 32643366 PMC7867030

[B17] Kavanagh K. Jornvall H. Persson B. Oppermann U. (2008). Medium- and short-chain dehydrogenase/reductase gene and protein families: the SDR superfamily: functional and structural diversity within a family of metabolic and regulatory enzymes. Cell. Mol. Life Sci. 65, 3895–3906. 10.1007/s00018-008-8588-y 19011750 PMC2792337

[B18] Kean K. M. Carpenter R. A. Pandini V. Zanetti G. Hall A. R. Faber R. (2017). High-resolution studies of hydride transfer in the ferredoxin: NADP(+) reductase superfamily. Febs J. 284, 3302–3319. 10.1111/febs.14190 28783258 PMC5626627

[B19] Kleckner I. R. Foster M. P. (2012). Guardd: user-friendly MATLAB software for rigorous analysis of CPMG RD NMR data. J. Biomol. Nmr 52, 11–22. 10.1007/s10858-011-9589-y 22160811 PMC3593345

[B20] Kovrigin E. L. Kempf J. G. Grey M. J. Loria J. P. (2006). Faithful estimation of dynamics parameters from CPMG relaxation dispersion measurements. J. Magnetic Reson. 180, 93–104. 10.1016/j.jmr.2006.01.010 16458551

[B21] Lisi G. P. Loria J. P. (2017). Allostery in enzyme catalysis. Curr. Opin. Struct. Biol. 47, 123–130. 10.1016/j.sbi.2017.08.002 28865247

[B22] McDonagh A. F. (2001). Turning green to gold. Nat. Struct. Biol. 8, 198–200. 10.1038/84915 11224558

[B23] Millet O. Loria J. P. Kroenke C. D. Pons M. Palmer A. G. (2000). The static magnetic field dependence of chemical exchange linebroadening defines the NMR chemical shift time scale. J. Am. Chem. Soc. 122, 2867–2877. 10.1021/ja993511y

[B24] Narayanan C. Bernard D. N. Bafna K. Gagne D. Chennubhotla C. S. Doucet N. (2018). Conservation of dynamics associated with biological function in an enzyme superfamily. Structure 26, 426–436.e3. 10.1016/j.str.2018.01.015 29478822 PMC5842143

[B25] Narayanan C. Gagne D. Reynolds K. A. Doucet N. (2017). Conserved amino acid networks modulate discrete functional properties in an enzyme superfamily. Sci. Rep. 7, 3207. 10.1038/s41598-017-03298-4 28600532 PMC5466627

[B26] Paukovich N. Xue M. J. Elder J. R. Redzic J. S. Blue A. Pike H. (2018). Biliverdin reductase B dynamics are coupled to coenzyme binding. J. Mol. Biol. 430, 3234–3250. 10.1016/j.jmb.2018.06.015 29932944 PMC6431292

[B27] Pereira P. J. B. Macedo-Ribeiro S. Parraga A. Perez-Luque R. Cunningham O. Darcy K. (2001). Structure of human biliverdin IXbeta reductase, an early fetal bilirubin IXbeta producing enzyme. Nat. Struct. Biol. 8, 215–220. 10.1038/84948 11224564

[B28] Quirk P. G. Jeeves M. Cotton N. P. J. Smith J. K. Jackson B. J. (1999). Structural changes in the recombinant, NADP(H)-binding component of proton translocating transhydrogenase revealed by NMR spectroscopy. Febs Lett. 446, 127–132. 10.1016/s0014-5793(99)00198-2 10100628

[B29] Ramberg H. Richardsen E. de Souza G. A. Rakaee M. Stensland M. E. Braadland P. R. (2021). Proteomic analyses identify major vault protein as a prognostic biomarker for fatal prostate cancer. Carcinogenesis 42, 685–693. 10.1093/carcin/bgab015 33609362 PMC8163044

[B30] Redzic J. S. Duff M. R. Blue A. Pitts T. M. Agarwal P. Eisenmesser E. Z. (2021). Modulating enzyme function via dynamic allostery within biliverdin reductase B. Front. Mol. Biosci. 8, 691208. 10.3389/fmolb.2021.691208 34095235 PMC8173106

[B31] Schlegel J. Armstrong G. S. Redzic J. S. Zhang F. L. Eisenmesser E. Z. (2009a). Characterizing and controlling the inherent dynamics of cyclophilin-A. Protein Sci. 18, 811–824. 10.1002/pro.89 19319933 PMC2762593

[B32] Schlegel J. Redzic J. S. Porter C. Yurchenko V. Bukrinsky M. Armstrong G. S. (2009b). Solution characterization of the extracellular region of CD147 and its interaction with its enzyme ligand cyclophilin-A. J. Mol. Biol. 391, 518–535. 10.1016/j.jmb.2009.05.080 19500591 PMC2940942

[B33] Shalloe F. Elliott G. Ennis O. Mantle T. J. (1996). Evidence that biliverdin-IX beta reductase and flavin reductase are identical. Biochem. J. 316, 385–387. 10.1042/bj3160385 8687377 PMC1217361

[B34] Sundaresan V. Yamaguchi M. Charton J. Stout C. D. (2003). Conformational change in the NADP(H) binding domain of transhydrogenase defines four states. Biochemistry 42, 12143–12153. 10.1021/bi035006q 14567675

[B35] Tsybovsky Y. Malakhau Y. Strickland K. C. Krupenko S. A. (2013). The mechanism of discrimination between oxidized and reduced coenzyme in the aldehyde dehydrogenase domain of Aldh1l1. Chemico-Biological Interact. 202, 62–69. 10.1016/j.cbi.2012.12.015 PMC3602205 23295222

[B36] Vagin A. Teplyakov A. (2010). Molecular replacement with MOLREP. Acta Crystallogr. D. Biol. Crystallogr. 66, 22–25. 10.1107/s0907444909042589 20057045

[B37] Vallurupalli P. Kay L. E. (2006). Complementarity of ensemble and single-molecule measures of protein motion: A relaxation dispersion NMR study of an enzyme complex. Proc. Natl. Acad. Sci. U. S. A. 103, 11910–11915. 10.1073/pnas.0602310103 16880391 PMC1567672

[B38] Vranken W. F. Boucher W. Stevens T. J. Fogh R. H. Pajon A. Llinas P. (2005). The CCPN data model for NMR spectroscopy: development of a software pipeline. Proteins-Structure Funct. Bioinforma. 59, 687–696. 10.1002/prot.20449 15815974

[B39] Whittier S. K. Hengge A. C. Loria J. P. (2013). Conformational motions regulate phosphoryl transfer in related protein tyrosine phosphatases. Science 341, 899–903. 10.1126/science.1241735 23970698 PMC4078984

[B40] Winter G. Waterman D. G. Parkhurst J. M. Brewster A. S. Gildea R. J. Gerstel M. (2018). Dials: implementation and evaluation of a new integration package. Acta Crystallogr. Sect. D-Structural Biol. 74, 85–97. 10.1107/s2059798317017235 PMC5947772 29533234

[B41] Wu S. Li Z. D. Gnatenko D. V. Zhang B. B. Zhao L. Malone L. E. (2016). BLVRB redox mutation defines heme degradation in a metabolic pathway of enhanced thrombopoiesis in humans. Blood 128, 699–709. 10.1182/blood-2016-02-696997 27207795 PMC4974201

