# Supplementary figures and images for: Identifying structural and dynamic changes during the Biliverdin Reductase B catalytic cycle
Source: Front Mol Biosci. 2023 Aug 14;10:1244587. doi: 10.3389/fmolb.2023.1244587 (PMC10461185; doi:10.3389/fmolb.2023.1244587)

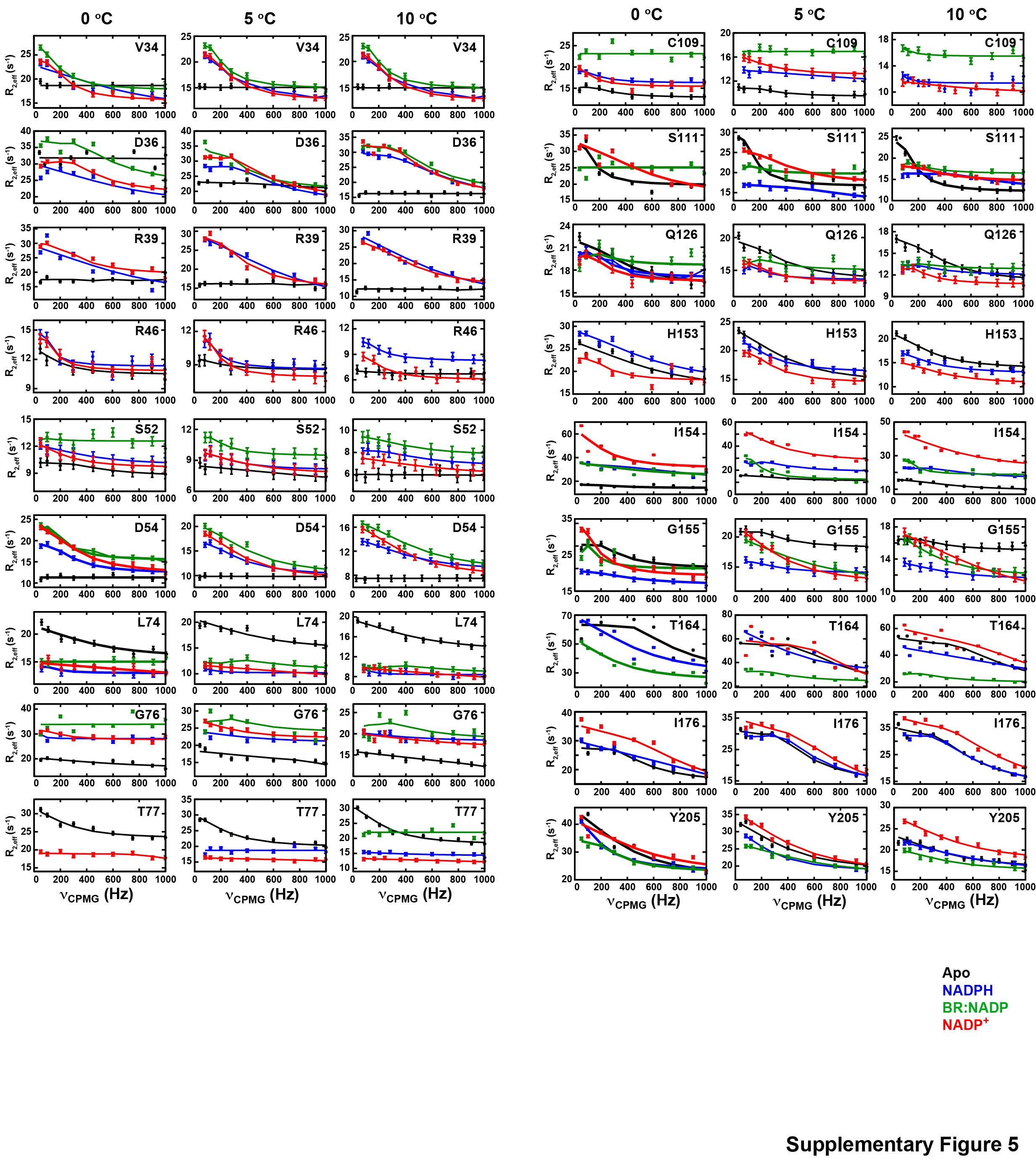

Supplement: Supplementary file 1 [file Image5.jpg]

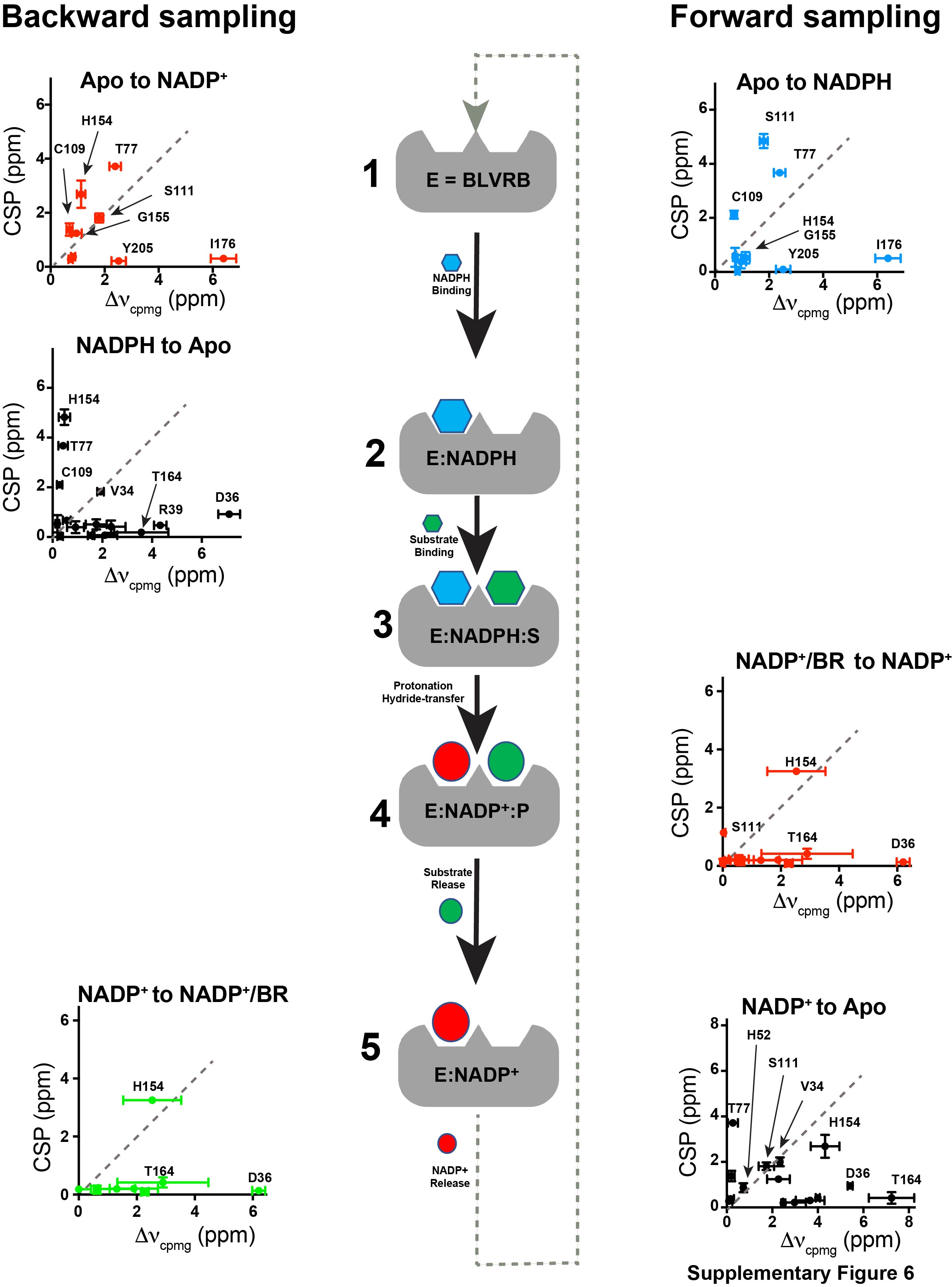

Supplement: Supplementary file 2 [file Image6.jpg]

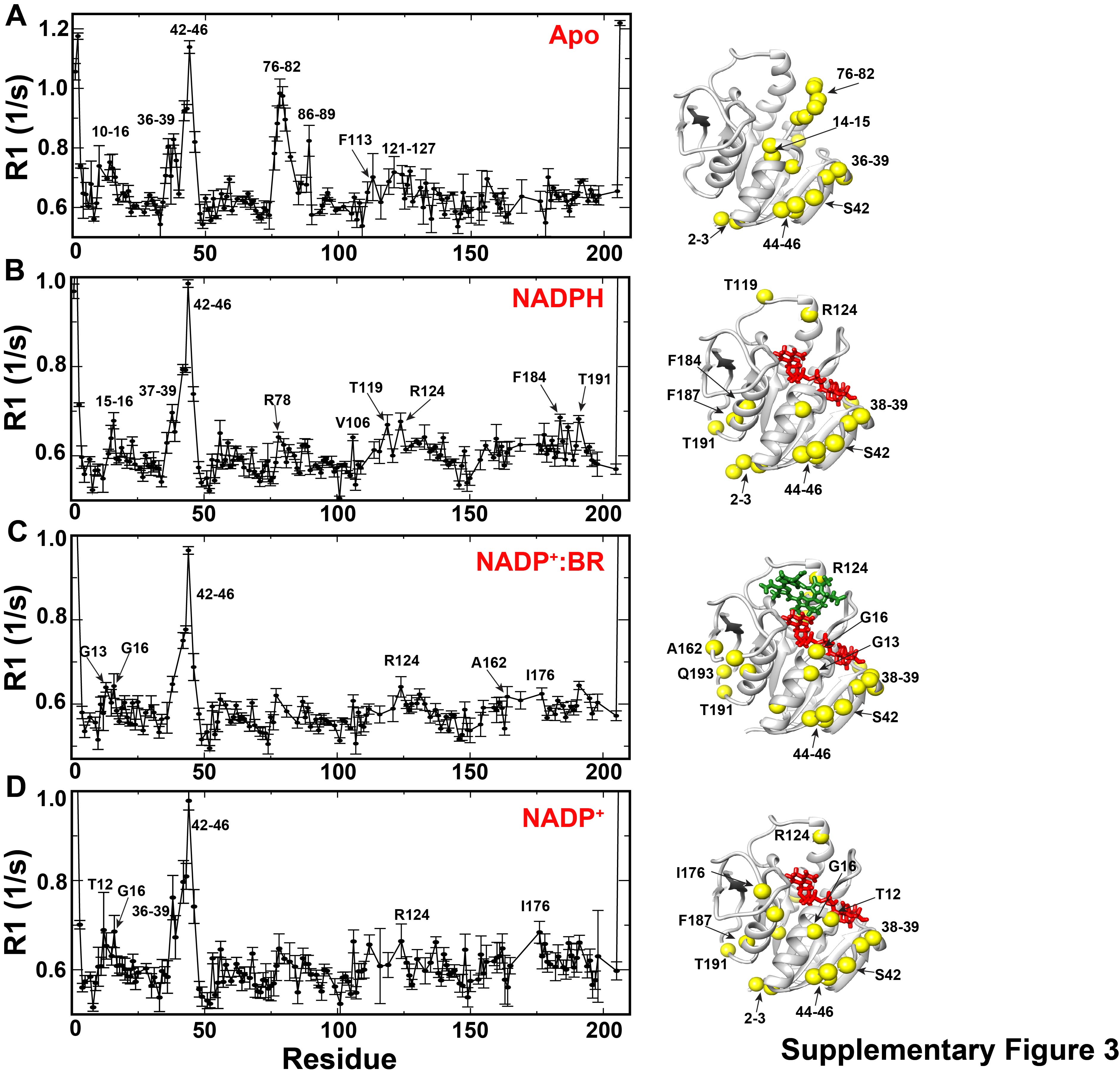

Supplement: Supplementary file 3 [file Image3.jpg]

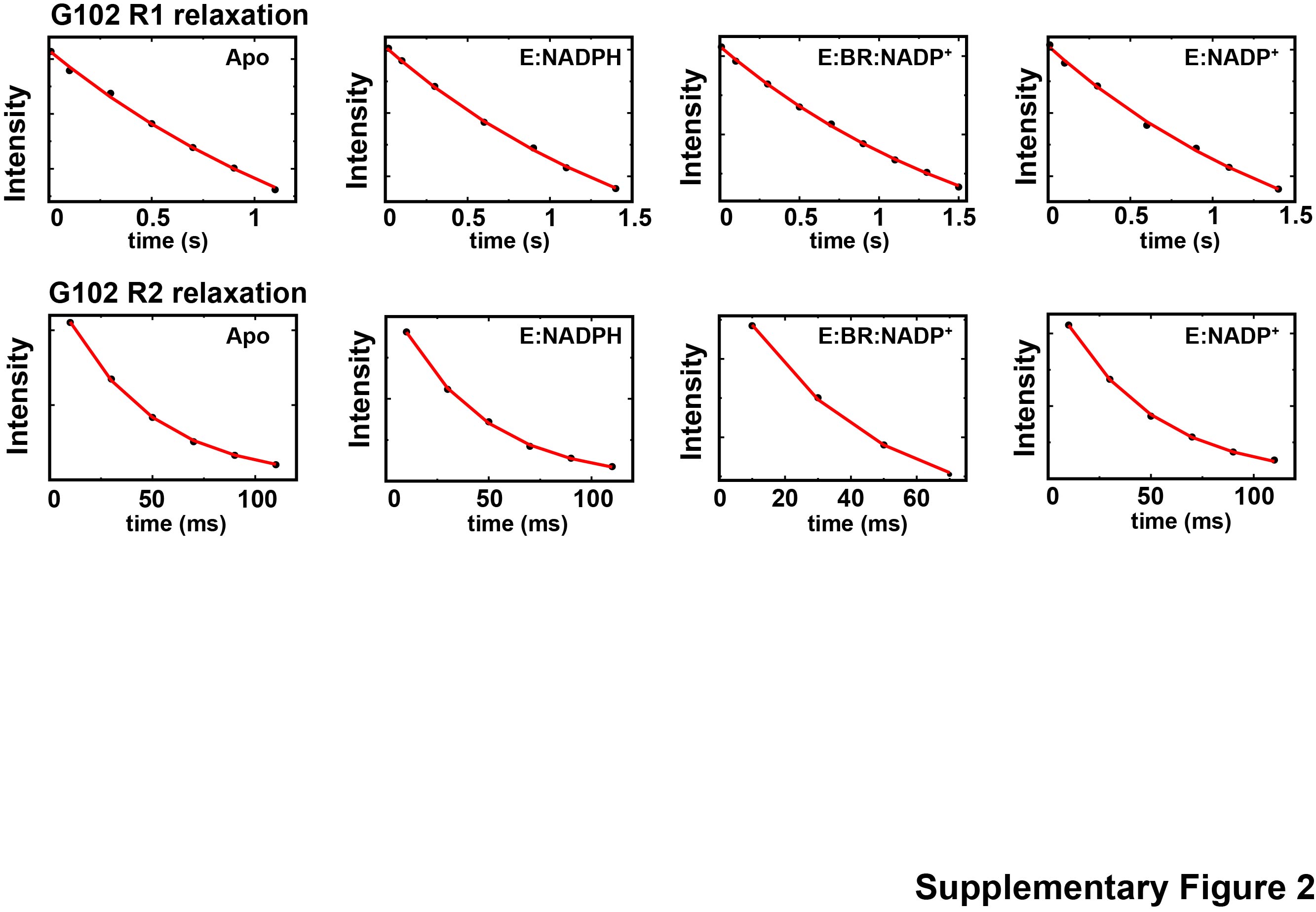

Supplement: Supplementary file 4 [file Image2.jpg]

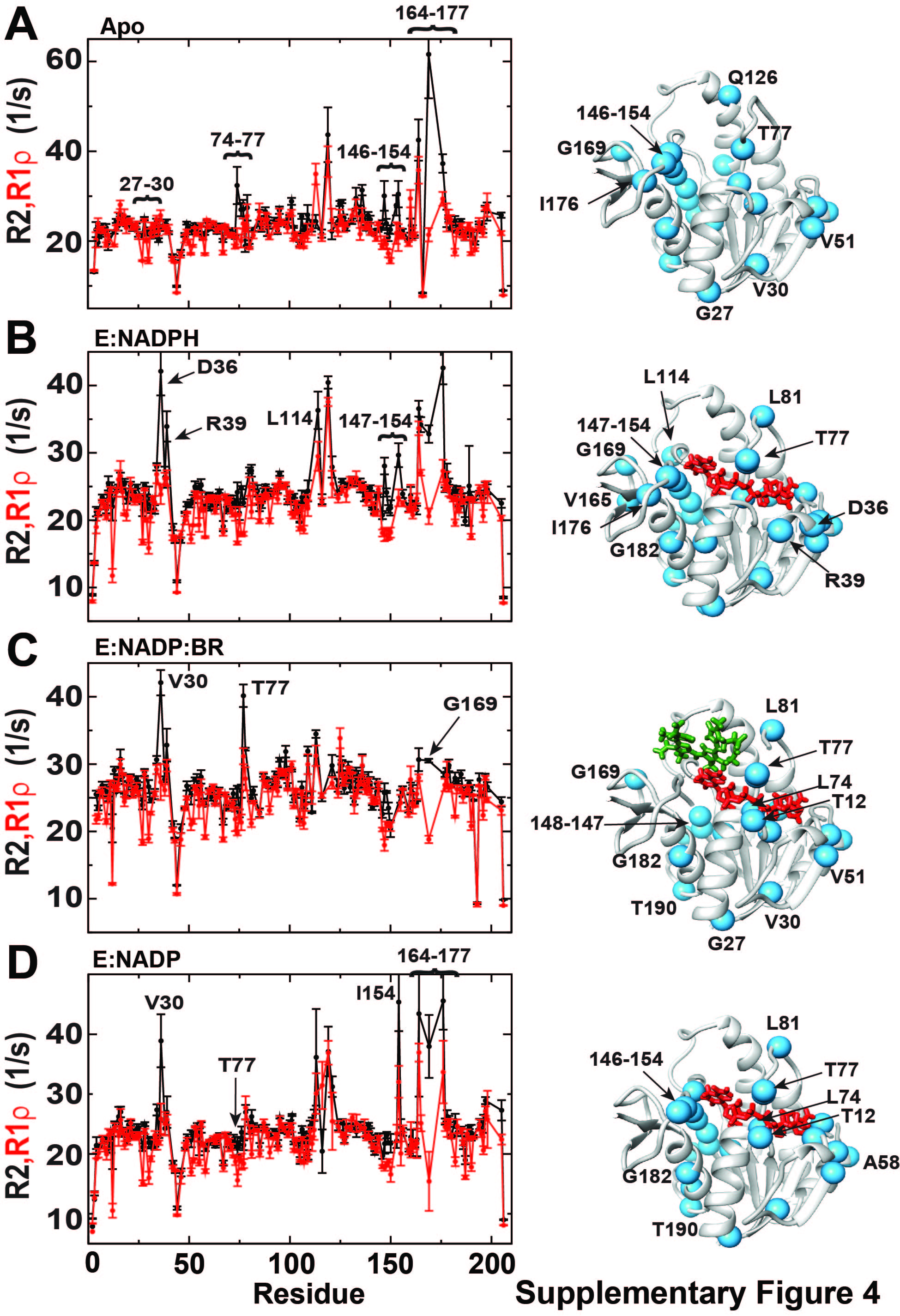

Supplement: Supplementary file 5 [file Image4.jpg]

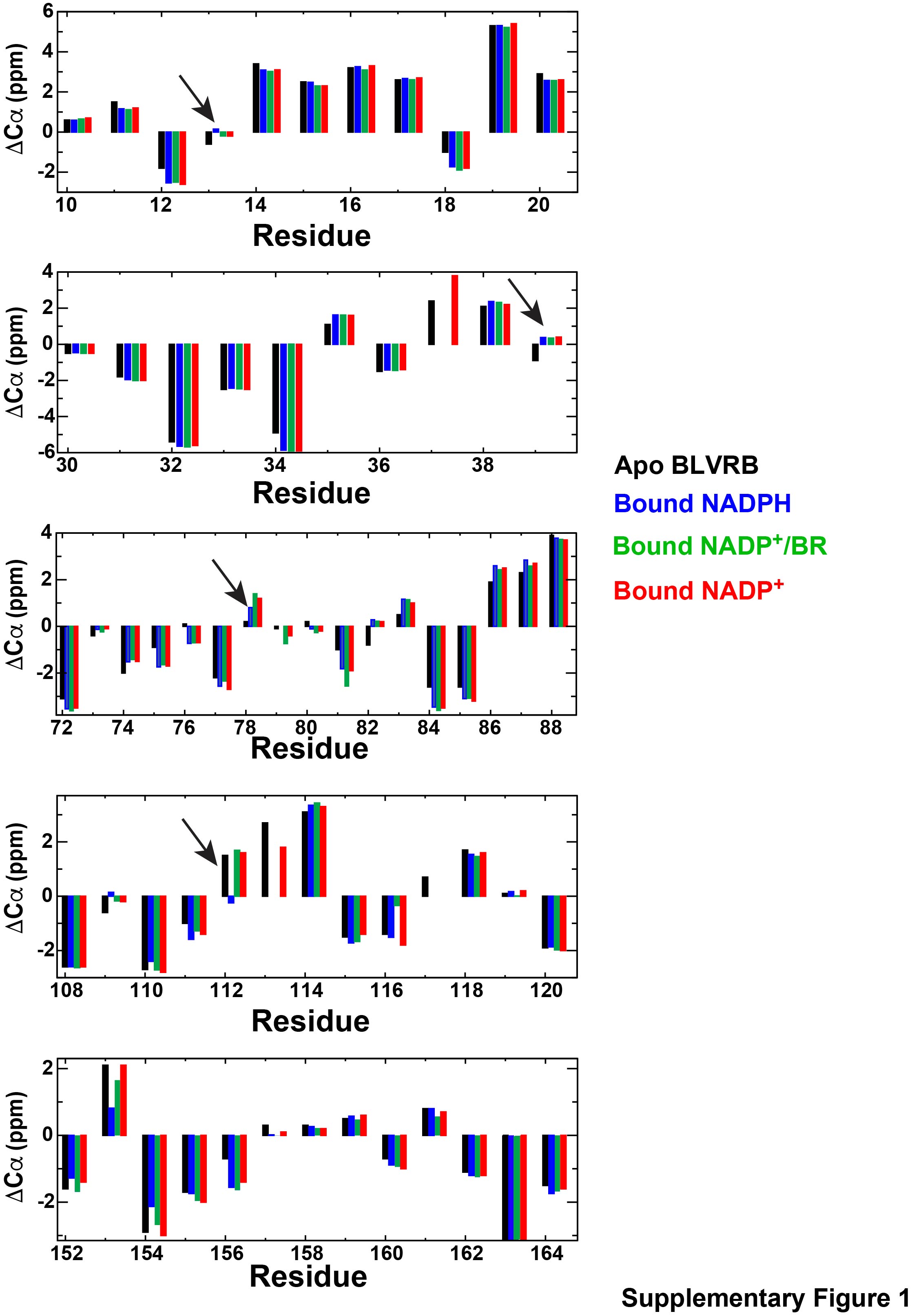

Supplement: Supplementary file 6 [file Image1.jpg]
